# A Highly Active Bidentate Magnesium Catalyst for Amine‐Borane Dehydrocoupling: Kinetic and Mechanistic Studies

**DOI:** 10.1002/chem.201901197

**Published:** 2019-04-25

**Authors:** Alexander C. A. Ried, Laurence J. Taylor, Ana M. Geer, Huw E. L. Williams, William Lewis, Alexander J. Blake, Deborah L. Kays

**Affiliations:** ^1^ School of Chemistry University of Nottingham University Park Nottingham NG7 2RD UK; ^2^ Centre for Biomolecular Sciences University of Nottingham University Park Nottingham NG7 2RD UK; ^3^ School of Chemistry The University of Sydney, F11 Eastern Ave Sydney NSW 2006 Australia; ^4^ Current address: Department of Chemistry University of Virginia Charlottesville Virginia 22904 USA

**Keywords:** amido ligands, dehydrocoupling, homogeneous catalysis, hydrogen storage, magnesium

## Abstract

A magnesium complex (**1**) featuring a bidentate aminopyridinato ligand is a remarkably selective catalyst for the dehydrocoupling of amine‐boranes. This reaction proceeds to completion with low catalyst loadings (1 mol %) under mild conditions (60 °C), exceeding previously reported s‐block systems in terms of selectivity, rate, and turnover number (TON). Mechanistic studies by in situ NMR analysis reveals the reaction to be first order in both catalyst and substrate. A reaction mechanism is proposed to account for these findings, with the high TON of the catalyst attributed to the bidentate nature of the ligand, which allows for reversible deprotonation of the substrate and regeneration of **1** as a stable resting state.

## Graphical abstract

**Reversible coordination**: A Mg^II^ complex featuring an aminopyridinato ligand is a remarkably selective catalyst for amine‐borane dehydrocoupling. The bidentate ligand allows the catalyst to reversibly regenerate the Mg^II^ complex as the resting state, rather than irreversibly forming a magnesium hydride species, leading to high efficiency and stability.

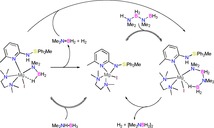

## Introduction

The transition from fossil fuels to a sustainable global energy system is one of the key challenges of the 21^st^ century. The increasing use of alternative sources has given rise to significant obstacles in storage and mobility; that is, how to utilize generated electricity for transportation or later use. Hydrogen is a promising option for a clean burning fuel, although issues remain over its storage.^1^, ^2^ Possible solutions include physical approaches such as high pressures, low temperatures and physisorption, as well as chemical storage in the form of hydrides.^3^, ^4^

Of these, amine‐boranes have emerged as lead candidates due to their high storage capacity, stability, and low environmental impact.^5^ These compounds have been the focus of considerable attention, with the presence of adjacent protic and hydridic hydrogens providing an excellent system for hydrogen storage and release.^6^, ^7^, ^8^ Hydrogen evolution from amine‐boranes may be achieved by solvolysis, thermolysis, catalysis, or some combination of the above. Of these, solvolysis creates an undesirable thermodynamic sink through the formation of very strong B−O bonds;^9^, ^10^ and thermolysis temperatures are often high, affording a complex mixture of products.^11^

In contrast, catalytic hydrogen release can occur at moderate temperatures, often yielding well‐defined products. Given the dominance of transition metals in catalysis, it is unsurprising that the d‐block has received the most attention in this field, with early dehydrocoupling catalysts based on rhodium and iridium.^12^, ^13^ Driven by considerations of resource availability and sustainability, recent investigations have focused on base metals such as titanium,^14^, ^15^ manganese,^16^, ^17^, ^18^, ^19^ iron,^20^, ^21^ cobalt,^22^, ^23^ and nickel.^5^

Looking beyond the transition metals, there has been significant work in recent years on catalysis with main group species.^24^, ^25^ This expansion has carried through to amine‐borane dehydrocoupling,^26^, ^27^, ^28^ with one of the most noteworthy examples being a *bis‐*(borane) reported by Wegner et al. capable of releasing 2.45 equivalents of H_2_ from NH_3_⋅BH_3_.^29^ Despite their ubiquitous nature, low cost, and low toxicity (barring beryllium and barium), the *s*‐block elements are underrepresented in this area. Predominantly investigated as storage materials rather than catalysts,^30^, ^31^, ^32^, ^33^ their lack of popularity may be due to poor selectivity and extended reaction times of, for example, 124 h (72 % conversion) with Me_2_NH⋅BH_3_ as a substrate, as reported for Group 1 *bis‐*(trimethylsilyl)amides.^34^ However, recent work is yielding more active systems.^35^, ^36^ Whilst Group 2 systems have shown greater activity towards more complex amine‐borane substrates,^37^, ^38^, ^39^ it should be noted that some systems studied have subsequently been shown to exhibit “spontaneous” dehydrocoupling.^40^ In contrast, the Me_2_NH⋅BH_3_ and *i*Pr_2_NH⋅BH_3_ substrates investigated here showed no hydrogen release in the absence of catalyst.

Herein, we report a highly active magnesium catalyst for the dehydrocoupling of Me_2_NH⋅BH_3_ and dehydrogenation of *i*Pr_2_NH⋅BH_3_. In addition to being readily synthesized on a multi‐gram scale, the catalyst exceeds previously published examples in terms of selectivity, substrate scope, turnover number (TON), and reaction rate. We present a detailed mechanistic investigation into this catalysis, including stoichiometric reactivity studies and kinetic measurements, and propose a catalytic cycle to account for these data.

## Results and Discussion

### Catalyst synthesis and investigation

**Scheme 1:**
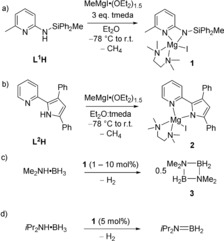
Synthesis of compounds **1** (a) and **2** (b), and catalytic dehydrocoupling/dehydrogenation of Me_2_NH⋅BH_3_ (c) and *i*Pr_2_NH⋅BH_3_ (d) by **1**.

Two magnesium complexes, **1** and **2**, were synthesized from the reaction of the proligands (**L^1^H** or **L^2^H**)^41^, ^42^ with MeMgI⋅(OEt_2_)_1.5_ and tetramethylethylenediamine (TMEDA) in diethyl ether (Scheme 1 a and b), and obtained in high yields and purity (**1**: 63 % yield; **2**: 86 % yield; see Supporting Information, Figures S2–S5, sections S3.2 and S4.1). The complexes were investigated as catalysts for the dehydrocoupling of Me_2_NH⋅BH_3_. While **2** was found to be unreactive, complex **1** gave excellent conversion of Me_2_NH⋅BH_3_ to the dimeric dehydrocoupling product [Me_2_NBH_2_]_2_ (**3**) with concomitant formation of H_2_ in C_6_D_6_ at 60 °C (Scheme 1 c, Figures S17–S18).

**Figure 1:**
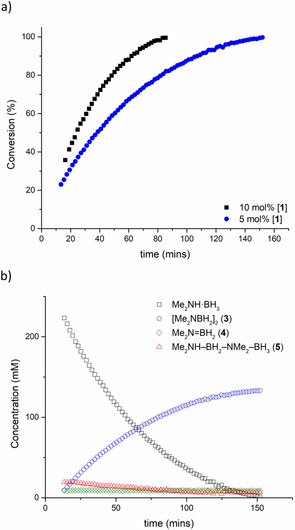
a) Conversion (mol %) vs. time (min) for the dehydrocoupling of Me_2_NH⋅BH_3_ with 5 mol % (•) and 10 mol % (▪) of **1** in C_6_D_6_ at 60 °C. Data obtained by in situ monitoring of the reaction by ^11^B NMR. b) Graph tracking concentration of substrate (Me_2_NH⋅BH_3_), product ([Me_2_NBH_2_]_2_; **3**), Me_2_N=BH_2_ (**4**), and Me_2_NH‐BH_2_‐NMe_2_‐BH_3_ (**5**) over reaction course. Concentrations determined by in situ ^11^B NMR measurements at 60 °C in C_6_D_6_. Initial concentrations [**3**]=0.29 m, [**1**]=15 mm.

**Figure 2:**
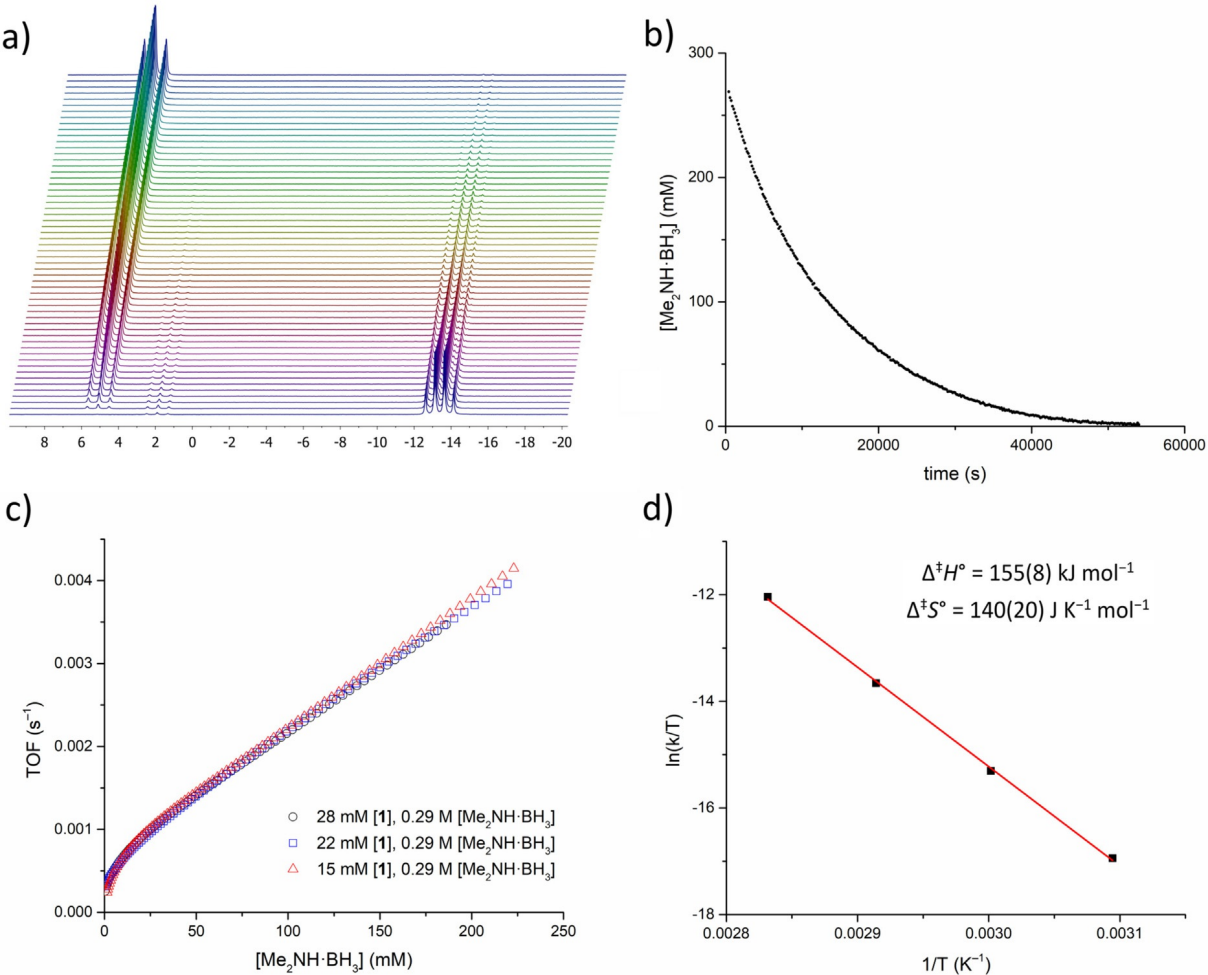
Mechanistic data from the dehydrocoupling of Me_2_NH⋅BH_3_ by **1**. a) Stacked ^11^B NMR spectra from in situ NMR measurements. b) Plot of [Me_2_NH⋅BH_3_] (mm, as determined by integration of ^11^B NMR spectra) vs. time for one set of kinetic data (toluene, 60 °C, 4.5 mm **1**). c) Plot of turnover frequency (TOF, υ⋅[**1**]^−1^) vs. concentration of Me_2_NH⋅BH_3_ (mm) for three experiments with different catalyst concentrations. d) Eyring plot for data collected over the temperature range 50–80 °C.

The reaction was monitored by in situ ^11^B NMR spectroscopy (Figure 2 a), discontinuous ^1^H and ^11^B NMR measurements (Figure S15), and volumetrically by the measurement of hydrogen evolution (Figures S25–S26). The reaction is remarkably selective, affording [Me_2_NBH_2_]_2_ (**3**) almost exclusively (<3 % Me_2_N=BH_2_, **4**, formed, Figure S18). High conversions are achieved in a relatively short time with low catalyst loadings (at 60 °C; ca. 99 % conversion after 80 mins at 10 mol % [**1**], 150 mins at 5 mol % [**1**]; Figure 1 a). High conversions were obtained even at catalyst loadings as low as 1 mol %, with >99 % in 60 h at 60 °C (implying a TON≥100; Figure S19). Increasing the temperature to 80 °C gives >95 % conversion in around 23 minutes with a catalyst loading of just 1.5 mol % (Figure S29d). The catalyst also works in THF, albeit at a significantly reduced rate (Figure S16), which is attributed to co‐ordination of the solvent to the catalyst active site.^43^ A similar solvent effect was seen with a lithium‐based dehydrocoupling catalyst studied by Mulvey and co‐workers.^44^

Reactions with the related amine‐borane *i*Pr_2_NH⋅BH_3_ afforded the dehydrogenated compound *i*Pr_2_N=BH_2_ as the dominant product (>95 %, Scheme 1 d). This reaction proceeded (albeit slowly) at room temperature (Figure S21 and S22) and increasing the temperature to 60 °C gave near total conversion in less than 1 hour (5 mol % catalyst loading, Figure S23). The formation of *i*Pr_2_N=BH_2_ has been observed in previous work on the catalytic dehydrogenation of *i*Pr_2_NH⋅BH_3_.^45^, ^46^, ^47^

To put this reactivity into context, the most effective previous alkaline earth catalyst for the reaction in Scheme 1 c was published by Hill et al. in 2010.^38^ This catalyst required 72 h at 60 °C to effect high (>90 %) conversions, and was less selective than **1**, affording several side products including (Me_2_N)_2_BH. The same catalyst proved more effective at amine‐borane dehydrocoupling with other substrates, catalyzing the reaction between secondary and primary amines with either pinacolborane (HBPin) or 9‐borabicyclo[3.3.1]nonane (9‐BBN).^48^ In most cases these reactions proceeded to completion in 1 h at room temperature (10 mol % catalyst loading).^48^ A related magnesium complex was effective at catalyzing the dehydrogenation of *i*Pr_2_NH⋅BH_3_ to *i*Pr_2_N=BH_2_, but could only effect stoichiometric conversions of Me_2_NH⋅BH_3_ to **3**.^47^ There have also been reports of magnesium and calcium bis(trimethylsilyl)amides catalyzing the dehydrocoupling of amine‐boranes with amines to afford asymmetric diaminoboranes.^49^ In most cases, however, these transformations required high temperatures and long reaction times (i.e. 70 °C, 48 h for coupling Me_2_NH⋅BH_3_ and *t*BuNH_2_, 2.5 mol % catalyst loading).^49^

Looking at Group 1 species, we can compare **1** to a recent lithium‐based catalyst published by Mulvey et al.^36^ Although relatively selective for the formation of [Me_2_NBH_2_]_2_ from Me_2_NH⋅BH_3_, the catalyst showed significantly lower activity than species **1**, with 89 % conversion after 60 h at 80 °C in toluene (2.5 mol % catalyst loading). Broadening our scope to the rare‐earth metals, a lanthanum hydride complex published by Okuda et al. catalyzed the dehydrocoupling of Me_2_NH⋅BH_3_ at a comparable rate to species **1** (100 % conversion in 2 h at 60 °C, 3 mol % catalyst loading).^50^ However, the catalyst was less selective than **1**, affording [Me_2_NBH_2_]_2_ and (Me_2_N)_2_BH in approximately 80:20 ratio.^50^ To the best of our knowledge, there are no examples of an alkaline earth catalyst (for any amine‐borane dehydrocoupling reaction) that are reported to be effective at <2.5 mol % loading,^39^ with **1** the first catalyst for which this has been demonstrated.^51^

Catalyst reusability was tested by repeated injections of Me_2_NH⋅BH_3_ to a solution of **1** in toluene, with reaction progress monitored by hydrogen evolution. At 10 mol % catalyst loading, **1** was found to tolerate two consecutive injections before a significant deterioration of activity was observed (Figure S26). This is likely due to catalyst decomposition from the gradual ingress of air and moisture with repeated injections. There are very few studies in the literature where the recyclability of main group catalysts for the dehydrocoupling of amine‐boranes has been ascertained,^29^ and to the best of our knowledge this is the first such determination for an *s*‐block metal catalyst.

Using the data gathered from in situ reaction monitoring by ^11^B NMR spectroscopy (Figures 1 and 2; Figures S27–S31), it is possible to gain significant mechanistic insight into this reaction. In addition to starting material, product (**3**), and trace amounts of Me_2_N=BH_2_ (**4**), the linear species Me_2_NH‐BH_2_‐NMe_2_‐BH_3_ (**5**) is also observed during the reaction.^52^ Compound **5** is a commonly observed intermediate in the dehydrocoupling of Me_2_NH⋅BH_3_,^14^, ^16^ and its concentration remains low throughout the reaction, gradually dropping to zero as the reaction nears completion (Figure 1 b). Compound **4** appears to form in the early stages of the reaction, and its concentration remains relatively constant throughout (Figure 1 b). Unlike **5**, its concentration does not drop to zero. Monitoring by ^1^H NMR reveals that **1** is regenerated at the end of the reaction, with no appreciable change in the signals arising from the catalyst (Figure S20).

Plotting reaction rate (υ) against substrate concentration [Me_2_NH⋅BH_3_] (Figure S28) indicates that the reaction is pseudo‐first order with respect to substrate at high concentrations, with a more complex rate dependence at low substrate concentrations.^53^ A plot of turnover frequency (TOF=υ⋅[**1**]^−1^) against substrate concentration for three different catalyst loadings shows overlay of the data sets (Figure 2 c), indicating that the reaction is first order with respect to catalyst.^53^ From this, a rate equation (valid for [Me_2_NH⋅BH_3_]≫[**1**]) of *υ*=*k*_obs_[Me_2_NH⋅BH_3_][**1**] has been derived.

Further measurements were carried out over a range of temperatures (50–80 °C) in toluene (Supporting Information, section S4.3.2). Extraction of the pseudo‐first order rate constants for these reactions (Figure S30) allowed for the construction of an Eyring plot (Figure 2 d) and determination of the activation parameters (Δ^≠^ *H*°=+155(8) kJ mol^−1^ and Δ^≠^ *S*°=+140(20) J K^−1^ mol^−1^).

Kinetic isotope effect (KIE) experiments were performed using deuterated substrate analogues. Reactions with Me_2_ND⋅BH_3_ proceeded at approximately the same rate (within error) as those with Me_2_NH⋅BH_3_, suggesting that N−D bond cleavage does not feature in the rate‐limiting step of the catalytic cycle. This stands in contrast to, for example, the iron‐catalyzed dehydrocoupling of Me_2_NH⋅BH_3_ reported by Webster et al., which displayed a KIE of 2.5±0.2 with Me_2_ND⋅BH_3_.^45^ The distinctive 1:1:1 triplet of HD was also observed by ^1^H NMR as the sole by‐product in reactions with Me_2_ND⋅BH_3_ (Figure S32). It was not possible to obtain a pure sample of Me_2_NH⋅BD_3_ from the literature procedure, which afforded mixtures of Me_2_NH⋅BD_3_ and Me_2_NH⋅BH_3_ (Supporting Information, section S4.1.4). The kinetic isotope effect for B−D bonds was thus calculated from an intermolecular competition experiment with this mixed sample,^54^ which gave a value of *k*_H_/*k*_D_=1.6±0.1 (Supporting Information, section 4.3.3). A mixture of H_2_ and HD was observed by ^1^H NMR spectroscopy in this reaction.

Stoichiometric reactions between Me_2_NH⋅BH_3_ and **1** (Supporting Information, section S4.4.1) resulted in the slow build‐up of **3**, **4**, **5**, and two additional signals at *δ*_B_=3.4 (t) and −14.6 (q) (Figure S35). This compound cannot be fully identified, but the signals are similar to previously reported magnesium complexes of amine‐boranes^38^, ^47^ and thus may correspond to an NMe_2_BH_2_NMe_2_BH_3_ chain bound to a magnesium atom. This compound is likely to be an intermediate of the catalytic cycle. Heating the sample to 60 °C resulted in the disappearance of these signals in the ^11^B NMR spectrum. Despite repeated attempts, no reaction intermediates containing coordinated magnesium could be crystallized from stoichiometric or substoichiometric experiments with Me_2_NH⋅BH_3_.

### Mechanistic discussion

**Scheme 2:**
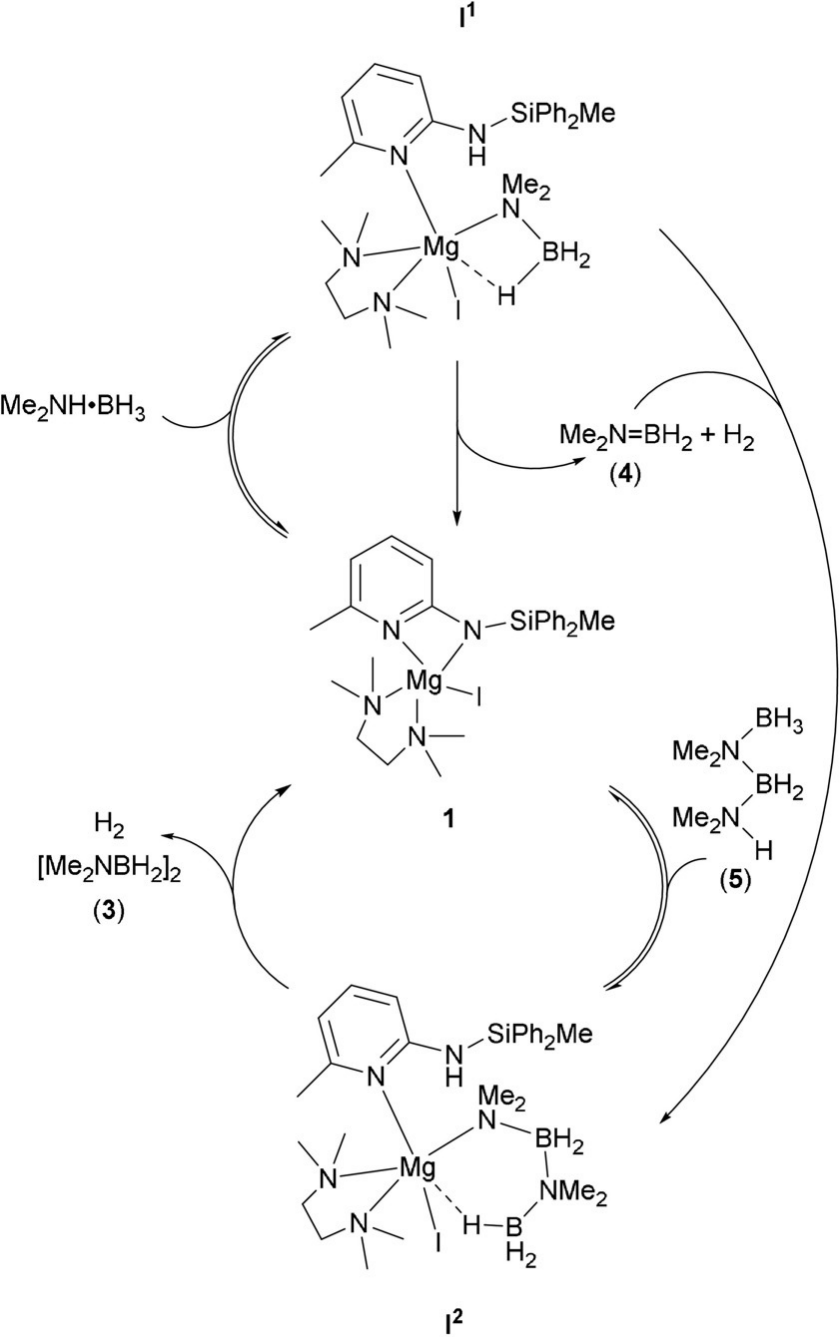
Proposed catalytic cycle for dehydrocoupling of Me_2_NH⋅BH_3_ by **1**.

We propose the catalytic cycle shown in Scheme 2 to account for the observed mechanistic data. This mechanism is broadly similar to those previously proposed for previous alkaline earth metal dehydrocoupling catalysts,^24^ but with some key differences due to the nature of the ligand **L^1^**. It should be noted that there has been little work on the kinetics of such systems, with the mechanism being inferred from the isolation of plausible intermediates and qualitative reaction monitoring by NMR spectroscopy.^38^, ^47^ As such, the kinetic data presented in this work provides useful supporting evidence for both this mechanism and the generalized mechanism of alkaline earth dehydrocoupling catalysts.

The first two steps involve reversible deprotonation of Me_2_NH⋅BH_3_ by the silylamide group of **1**, to give species **I^1^**, followed by an irreversible β‐hydride elimination; affording hydrogen, **4**, and species **1**. This step likely proceeds via a short‐lived magnesium hydride, which rapidly deprotonates the pendant silyl amine to regenerate **1**. This mechanism is somewhat different to previous alkaline earth catalysts, which were added as pre‐catalysts and underwent an initial irreversible deprotonation reaction, typically with elimination of an alkyl or amino species.^24^ Here, because of the bidentate nature of **L^1^**, the amine generated is held proximal to the magnesium and continues to take part in the reaction. This also helps explain the inactivity of **2** as a catalyst, as the less basic pyrrolide is unlikely to deprotonate Me_2_NH⋅BH_3_.

Species **I^1^** can also undergo an insertion reaction with **4** to generate species **I^2^**. This intermediate is considered to correspond to the unknown species observed by ^11^B NMR in the stoichiometric experiments (Figure S35), and similar insertion reactions are commonly invoked in mechanisms of this type.^24^ While it has not been possible to isolate the intermediates **I^1^** and **I^2^**, we consider them plausible based on previously proposed mechanisms for similar catalysts.^24^, ^38^ Furthermore, the silylamide is the only group on the complex sufficiently basic to deprotonate the substrate. The partial dissociation of the ligand, with the pyridine nitrogen remaining bound to the magnesium atom, seems a reasonable explanation for the regeneration of **1** at the end of the reaction with no loss of ligand. Species **I^2^**, once formed, can undergo either a reversible proton exchange (affording linear intermediate **5**) or an irreversible δ‐hydride elimination to afford the major product (**3**) and hydrogen.

**Scheme 3:**
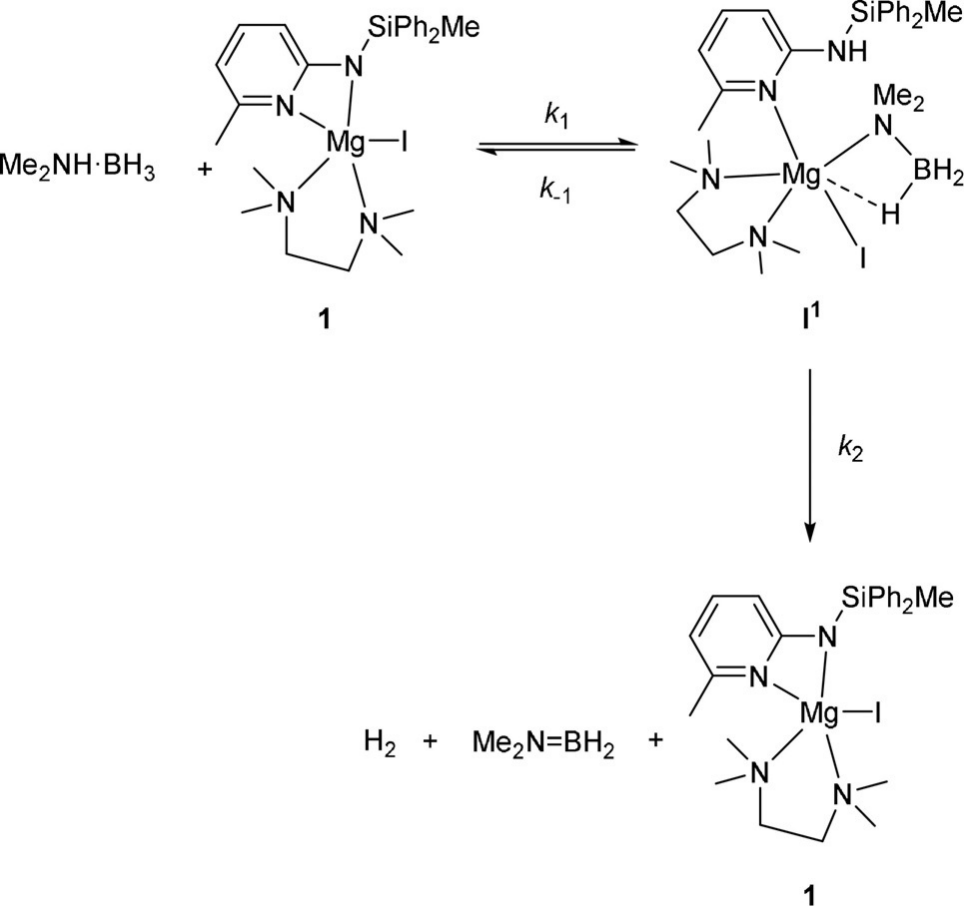
Rate controlling steps of the proposed catalytic cycle.

**Figure 3:**
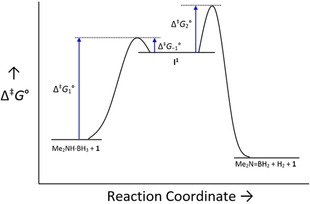
Schematic potential energy surface (PES) for the first two steps of the proposed catalytic cycle.

Based on the first order dependence on both **1** and Me_2_NH⋅BH_3_, the first two steps of the proposed mechanism (formation of **I^1^** and subsequent β‐hydride elimination) must control the overall rate of the catalytic cycle. This is best modelled using the pre‐equilibrium approximation,^55^ in which an equilibrium is established between (Me_2_NH⋅BH_3_+**1**) and **I^1^**, followed by a slow β‐hydride elimination (Scheme 3). A schematic potential energy surface is shown in Figure 3 for illustrative purposes. Denoting the rate constants as *k*_1_, *k*_‐1_, and *k*_2_ (Scheme 3); it can be shown that the overall rate equation for this process is given by Equation (1):^55^

(1) $$v=\frac{k{}_{1}k{}_{2}}{k{}_{-1}}\left[\mathrm{Me}{}_{2}\mathrm{NH}\cdot \mathrm{BH}{}_{3}\right]$$

Use of Me_2_ND⋅BH_3_ as a substrate in this reaction will reduce the rate of both *k*_1_ and *k*_−1_. As a result, the overall rate of reaction does not change significantly. This can also be understood as the overall rate of reaction being determined by the position of equilibrium for this deprotonation, rather than the rate of deprotonation. By contrast, use of Me_2_NH⋅BD_3_ will reduce the rate of *k*_2_, resulting in a modest KIE for this substrate. The fact that β‐hydride elimination is the rate‐limiting step (as indicated by the KIE) suggests that the initial deprotonation must be a reversible process. If it were irreversible, one would expect intermediate **I^1^** to accumulate in the reaction, which is not observed.

Because of the pre‐equilibrium in this reaction, the overall kinetic energy barrier for this process (Δ^≠^ *G*°) will depend on the energy barriers for *k*_1_, *k*_−1_, and *k*_2_ according to Equation (2):^55^

(2) $$\Delta {}^{\neq }G{}^{\circ }=\Delta {}^{\neq }G{}_{1}{}^{\circ }+\Delta {}^{\neq }G{}_{2}{}^{\circ }-\Delta {}^{\neq }G{}_{-1}{}^{\circ }$$

This is shown visually with the schematic potential energy surface in Figure 3. This means that the experimentally determined activation parameters for this reaction will depend on all three of these processes. As Δ^≠^ *G*_1_° is the largest single term in this expression (Figure 3), the activation parameters will be dominated by the transition state for the deprotonation step, even though the β‐hydride elimination is the slowest step. This results in the large positive entropy of activation (Δ^≠^ *S*° = +140(20) J K^−1^ mol^−1^, consistent with partial dissociation of the bidentate ligand) and large enthalpy of activation (Δ^≠^ *H*° = +155(8) kJ mol^−1^, consistent with significant bond cleavage as we approach the transition state). It should be noted that similar activation parameters were obtained for the dehydrocoupling of 9‐BBN and *N*‐methylaniline by Hill's magnesium catalyst (Δ^≠^ *H*° = +125.1(3) kJ mol^−1^ and Δ^≠^ *S*° = +85(2) J K^−1^ mol^−1^), and that this was also attributed to deprotonation of the amine.^48^

The observed deviation from first‐order kinetics in Me_2_NH⋅BH_3_ can be explained by the insertion reaction between **I^1^** and **4** being non‐rate limiting provided [Me_2_NH⋅BH_3_]≫[**1**], and becoming rate limiting as the reaction nears completion. The fact that compound **5** is fully consumed at the end of the reaction, while some **4** remains unreacted, can be explained by both species being steady‐state intermediates of the catalytic cycle (i.e. their rate of formation and consumption is approximately equal). This means the concentration of both **4** and **5** remains low and relatively constant throughout. As the reaction nears completion, **5** is fully converted to **3** through reaction with the catalyst. However, compound **4** can only be consumed in the presence of both Me_2_NH⋅BH_3_ and **1** (see Scheme 2). As the reaction nears completion, all the Me_2_NH⋅BH_3_ will be converted to **4** by reacting with the catalyst. This means that there is no Me_2_NH⋅BH_3_ left for **4** to react with, and thus a small amount of **4** is left unreacted once all substrate is consumed. It should be noted that the off‐metal dimerization of **4** to **3** has been observed previously, and may occur in our system given sufficient time.^56^, ^57^, ^58^ However, we did not observe this, and it is likely that this second‐order process is slow in the catalytic regime.^47^ Finally, for the reaction of *i*Pr_2_NH⋅BH_3_ with **1**, it is probable that the insertion reaction is prevented by the increased steric bulk, leading to *i*Pr_2_N=BH_2_ as the major product. Thus, the mechanism shown in Scheme 2 accounts for all the observed mechanistic data; including the rate dependencies, kinetic isotope effects, activation parameters, and observed intermediates.

## Conclusions

Magnesium complex **1** is an effective catalyst for the dehydrocoupling/dehydrogenation of Me_2_NH⋅BH_3_ and *i*Pr_2_NH⋅BH_3_. The dehydrocoupling of Me_2_NH⋅BH_3_ afforded [Me_2_NBH_2_]_2_ more cleanly, rapidly, and at lower catalyst loadings than any previously reported alkaline earth metal catalyst. We propose that the bidentate ligand **L^1^** allows the catalyst to reversibly regenerate **1** as the catalytic resting state, rather than irreversibly forming a magnesium hydride species. This distinguishes the system from previous catalysts and may account for the remarkably high efficacy and stability of **1** as a catalyst for amine‐borane dehydrocoupling.

## Experimental Section

All experimental details including synthetic methods, catalytic studies, kinetic measurements, and crystallographic data are provided in the Supporting Information.

## Conflict of interest

The authors declare no conflict of interest.

## Supporting information

As a service to our authors and readers, this journal provides supporting information supplied by the authors. Such materials are peer reviewed and may be re‐organized for online delivery, but are not copy‐edited or typeset. Technical support issues arising from supporting information (other than missing files) should be addressed to the authors.

Supplementary
