# Supplementary material for: A Highly Active Bidentate Magnesium Catalyst for Amine‐Borane Dehydrocoupling: Kinetic and Mechanistic Studies
Source: Chemistry. 2019 Apr 25;25(27):6840–6. doi: 10.1002/chem.201901197 (PMC6563444; doi:10.1002/chem.201901197)
Supplement: Supplementary file 1 — Supplementary [file CHEM-25-6840-s001.pdf]

CHEMISTRY
A European Journal

Supporting Information

A Highly Active Bidentate Magnesium Catalyst for Amine-Borane Dehydrocoupling: Kinetic and Mechanistic Studies

Alexander C. A. Ried+,[a] Laurence J. Taylor+,[a] Ana M. Geer+,[a, d] Huw E. L. Williams,[b] William Lewis,[c] Alexander J. Blake,[a] and Deborah L. Kays*[a]

chem_201901197_sm_miscellaneous_information.pdf

Contents

S1 – General Procedures 2

S2 – Instrumentation 2

S3 – Crystallography 2

S3.1 – Crystallographic Methods 2

S3.2 – Crystallographic Data 3

S4 – Experimental Procedures 4

S4.1 – Synthetic Protocols 4

S4.1.1 – Synthesis of 1 ($L^1MgI·(tmeda)$) 4

S4.1.2 – Synthesis of 2 ($L^2MgI·(tmeda)$) 6

S4.1.3 – Synthesis of $Me_2ND·BH_3$ 8

S4.1.4 – Synthesis of $Me_2NH·BD_3$ 8

S4.2 – Reaction Monitoring 13

S4.2.1 – Standard procedure for the catalytic dehydrocoupling of $Me_2NH·BH_3$ 13

S4.2.2 – Qualitative *in situ* monitoring by ^1H NMR spectroscopy 17

S4.2.3 – Standard procedure for the catalytic dehydrocoupling of *i*$Pr_2NH·BH_3$ 18

S4.2.4 – Hydrogen Evolution Experiments 20

S4.3 – Kinetic Experiments 22

S4.3.1 – Procedure for determining reaction orders 22

S4.3.2 – Procedure for determining activation parameters 24

S4.3.3 – Kinetic Isotope Effect with $Me_2ND·BH_3$ 27

S4.3.3 – Kinetic Isotope Effect with $Me_2NH·BD_3$ 29

S4.4 – Stoichiometric Experiments 30

S4.4.1 – Reaction with $Me_2NH·BH_3$ 30

S4.4.2 – Reaction with $Me_3N·BH_3$ 31

S6 – References 32

S1 – General Procedures

All manipulations involving magnesium complexes **1** and **2** were conducted under anhydrous, anaerobic conditions using standard Schlenk line and glove box techniques. Standard laboratory solvents were dried by distilling from potassium (toluene) or sodium-benzophenone ketyl (THF) and stored over a potassium mirror (toluene) or 4 Å molecular sieves (THF). d_6-Benzene was dried over potassium in a sealed ampoule at 80 °C for 4 days, before vacuum-transferring to a Young's flask containing a potassium mirror, which was subsequently stored in the glovebox prior to use. NMR samples of air and moisture sensitive compounds were prepared using glove box techniques and contained in Young´s tap modified borosilicate glass NMR tubes. TMEDA was distilled from CaH_2 and stored over 4 Å molecular sieves. $Me_2NH{\cdot}BH_3$, $Me_3N{\cdot}BH_3$, and $i$$Pr_2NH{\cdot}BH_3$ were purchased from Sigma-Aldrich and used as received. Ligand precursors **L¹H** and **L²H** were synthesised by minor modifications of previous reported synthetic procedures.[1,2] $MeMgI{\cdot}(OEt_2)_{1.5}$ was synthesised from the reaction between activated magnesium turnings and iodomethane in diethyl ether. Purified compounds were stored under dried nitrogen in an MBraun UNIlab glovebox.

S2 – Instrumentation

All NMR data was collected on Bruker DPX300, DPX400, AV400, AV(III)400, AV(III)400HD or AV(III)600 spectrometers. Chemical shifts are quoted in ppm relative to TMS (^{1}H, ^{13}C{^{1}H}) and $BF_3{\cdot}OEt_2$ (^{11}B, ^{11}B{^{1}H}). ^{11}B NMR spectra used for quantitative analysis were processed with linear back prediction to eliminate signals arising from borosilicate glass.

S3 – Crystallography

S3.1 – Crystallographic Methods

Under a flow of N_2, crystals suitable for X-ray diffraction were quickly removed from the crystallisation vessel and covered with Fomblin® (YR-1800 perfluoropolyether oil). A suitable crystal was then mounted on a polymer-tipped MicroMount™ and cooled rapidly to 120 K in a stream of cold N_2 using an Oxford Cryosystems open flow cryostat.[3] Single crystal X-ray diffraction data were collected on an Oxford Diffraction SuperNova Duo diffractometer (Atlas CCD area detector, mirror-monochromated Cu-$K\alpha$ radiation source; λ = 1.54184 Å or mirror-monochromated Mo-$K\alpha$ radiation source; λ = 0.71073 Å; ω scans). Absorption corrections were applied using an analytical numerical method (CrysAlis Pro).[4] All non-H atoms were located using direct methods[5] and difference Fourier syntheses. Hydrogen atoms were placed and refined using a geometric riding model. All fully occupied non-H atoms were refined with anisotropic displacement parameters, unless otherwise specified. Crystal structures were solved and refined using the Olex2 software package.[6,7] Programs used include CrysAlisPro[8] (control of Supernova, data integration and absorption correction), SHELXL[9] (structure refinement), SHELXS[5] (structure solution), SHELXT[10] (structure solution), OLEX2[6] (molecular graphics). CIF files were checked using checkCIF[11] CCDC-1836622 and -1836623 contain the supplementary data for **1** and **2**. These data can be obtained free of charge from The Cambridge Crystallographic Data Centre *via* www.ccdc.cam.ac.uk/data_request/cif.

S3.2 – Crystallographic Data

Crystal Data for 1. Crystal Data for $C_{25}H_{35}IMgN_4Si$ (M_r = 570.87 g/mol): triclinic, space group *P*-1 (no. 2), a = 10.5405(3) Å, b = 15.3220(6) Å, c = 17.2414(7) Å, α = 93.029(3)°, β = 96.647(3)°, γ = 91.904(3)°, V = 2759.75(17) Å^3, Z = 4, T = 120(2) K, μ(Cu$K\alpha$) = 9.891 mm^{-1}, D_{calc} = 1.374 g/cm^3, 25950 reflections measured (7.534° ≤ 2Θ ≤ 133.192°), 9758 unique (R_{int} = 0.0565, R_{sigma} = 0.0502) which were used in all calculations. The final R_1 was 0.0837 ($I > 2\sigma(I)$) and wR_2 was 0.2446 (all data).

Crystal Data for 2. Crystal Data for $C_{29}H_{36}IMgN_4O_{0.5}$ (M_r = 599.83 g/mol): monoclinic, space group $P2_1/n$ (no. 14), a = 9.8891(5) Å, b = 40.968(2) Å, c = 13.9555(6) Å, β = 91.158(3)°, V = 5652.8(5) Å^3, Z = 8, T = 120(2) K, μ(Cu$K\alpha$) = 9.310 mm^{-1}, D_{calc} = 1.410 g/cm^3, 14475 reflections measured (7.666° ≤ 2Θ ≤ 148.54°), 8968 unique (R_{int} = 0.0271, R_{sigma} = 0.0300) which were used in all calculations. The final R_1 was 0.0570 ($I > 2\sigma(I)$) and wR_2 was 0.1564 (all data).

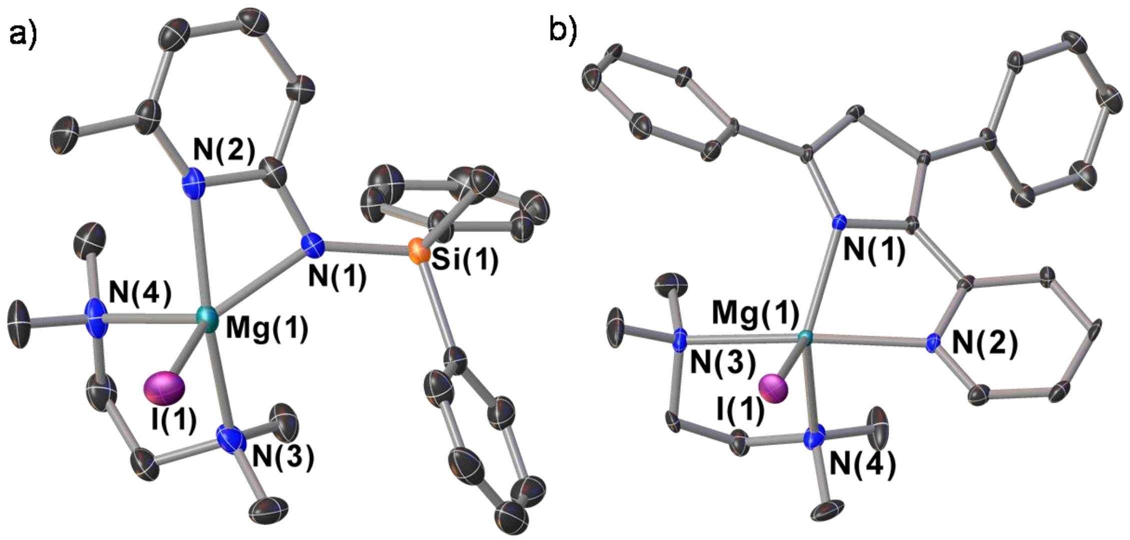

Figure S1: Molecular structure of **1** (a) and **2** (b) with anisotropic displacement ellipsoids set at 50% probability. Hydrogen atoms, second molecule in asymmetric unit (**1**, **2**), and co-crystallised diethyl ether (**2**) have been omitted for clarity.

Table S1: Selected distances (Å) and angles (°) for **1** and **2**. Measurements for second molecule in asymmetric unit in square brackets.

**1**			
Mg(1)–N(1)	2.082(8) [2.073(7)]	Mg(1)–N(2)	2.180(9) [2.198(9)]
Mg(1)–N(3)	2.296(9) [2.27(1)]	Mg(1)–N(4)	2.217(8) [2.239(8)]
Mg(1)–I(1)	2.745(3) [2.744(3)]	N(1)–Mg(1)–N(2)	64.3(3) [64.2(3)]
N(3)–Mg(1)–N(4)	80.1(3) [79.7(3)]		
**2**			
Mg(1)–N(1)	2.102(4) [2.078(4)]	Mg(1)–N(2)	2.189(4) [2.185(4)]
Mg(1)–N(3)	2.308(4) [2.296(4)]	Mg(1)–N(4)	2.270(4) [2.252(5)]
Mg(1)–I(1)	2.721(2) [2.732(2)]	N(1)–Mg(1)–N(2)	76.9(2) [75.8(2)]
N(3)–Mg(1)–N(4)	79.3(2) [79.1(2)]		

S4 – Experimental Procedures

S4.1 – Synthetic Protocols

S4.1.1 – Synthesis of 1 (L^1MgI·(tmeda))

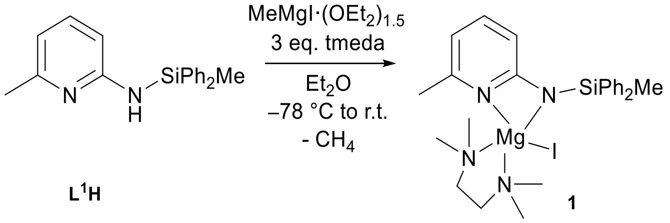

Scheme S1: Synthesis of compound **1**

To a suspension of MeMgI·$(Et_2O)_{1.5}$ (1.526 g, 5.5 mmol) in diethyl ether (50 mL) at −78 °C was added dropwise a solution of **L^1H** (10 mL, 0.5 M in Et_2O/hexanes, 5 mmol) over 45 min. The solution was stirred at −78 °C for a further 2 h, then allowed to warm to −30 °C. TMEDA (2.25 mL, 15 mmol) was added and the resultant gel warmed to room temperature overnight. The milky suspension was filtered, and volatiles removed from the filtrate *in vacuo*; washing of the residue with cold hexanes afforded compound **1** as a white solid (1.80 g, 63%). Crystals of **1** suitable for X-ray diffraction were grown from a saturated solution of the complex in diethyl ether at –30 °C.

^{1}H NMR (C_6D_6, 25 °C, 400 MHz): δ = 0.73 (s, 3H, Si*CH*$_3$), 1.63 (s, 4H, *tmeda*), 1.77 (s, 12H, *tmeda*), 2.39 (s, 3H, Py*CH*$_3$), 5.98 (dt, *J* = 7.1, 0.7 Hz, 1H, Py*H*), 6.21 (dt, *J* = 8.4, 0.8 Hz, 1H, Py*H*), 6.80 (dd, *J* = 8.4, 7.1 Hz, 1H, Py*H*), 7.19 (tt, J = 7.4, 1.4 Hz, 2H, $SiPh_2^p$), 7.28 (tt, J = 7.9, 1.4 Hz, 4H, $SiPh_2^m$), 7.87 (dd, *J* = 8.0, 1.4 Hz, 4H, $SiPh_2^o$).

$^{13}C\{^1H\}$ NMR (C_6D_6, 25 °C, 101 MHz): δ = −0.1 (Si(*C*H$_3$)Ph$_2$), 23.3 (Py*C*H$_3$), 46.8 (*tmeda*), 56.1 (*tmeda*), 109.7 (Py), 111.6 (Py), 128.9 (Ph^m), 129.2 (Ph^p), 135.0 (Ph^o), 138.4 (Py), 140.8 (Ph^i), 154.5 (Py), 170.1 (Py).

$C_{25}H_{35}IMgN_4Si$ (570.15): calc'd (%) C 52.60, H 6.18, N 9.81; found (%) C 52.49, H 6.03, N 9.72.

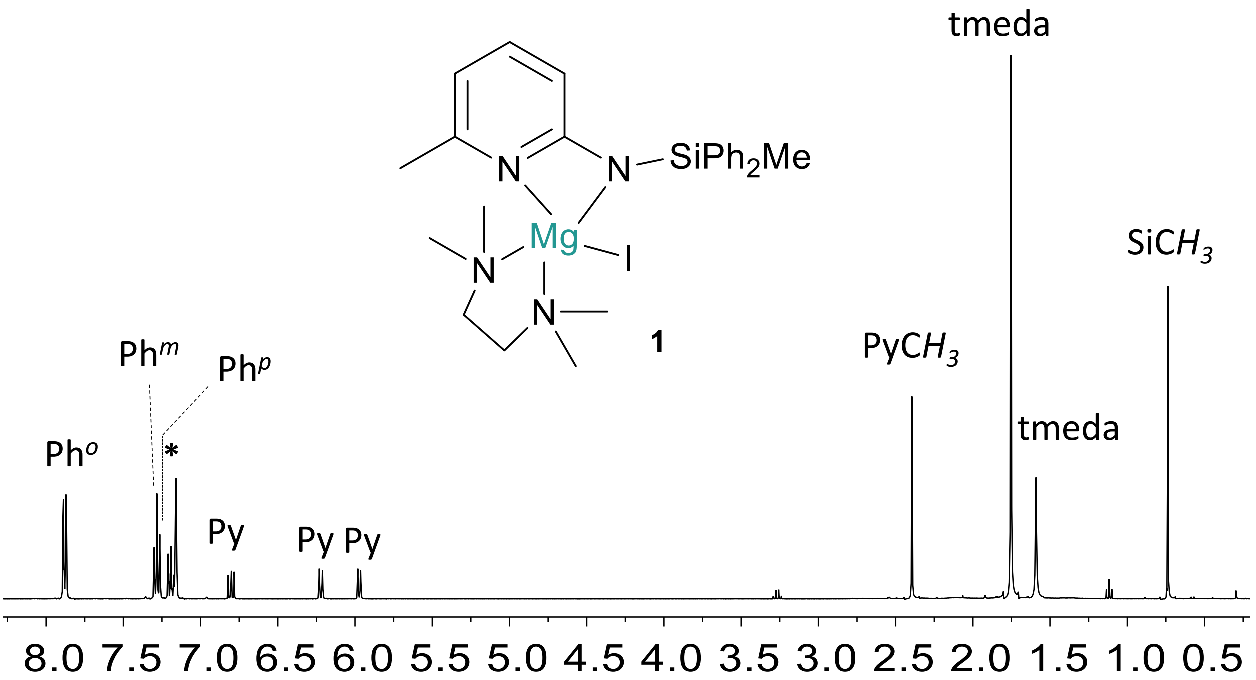

Figure S2: ^{1}H NMR (400 MHz) spectra of **1** in C_6D_6 (*) at 25 °C.

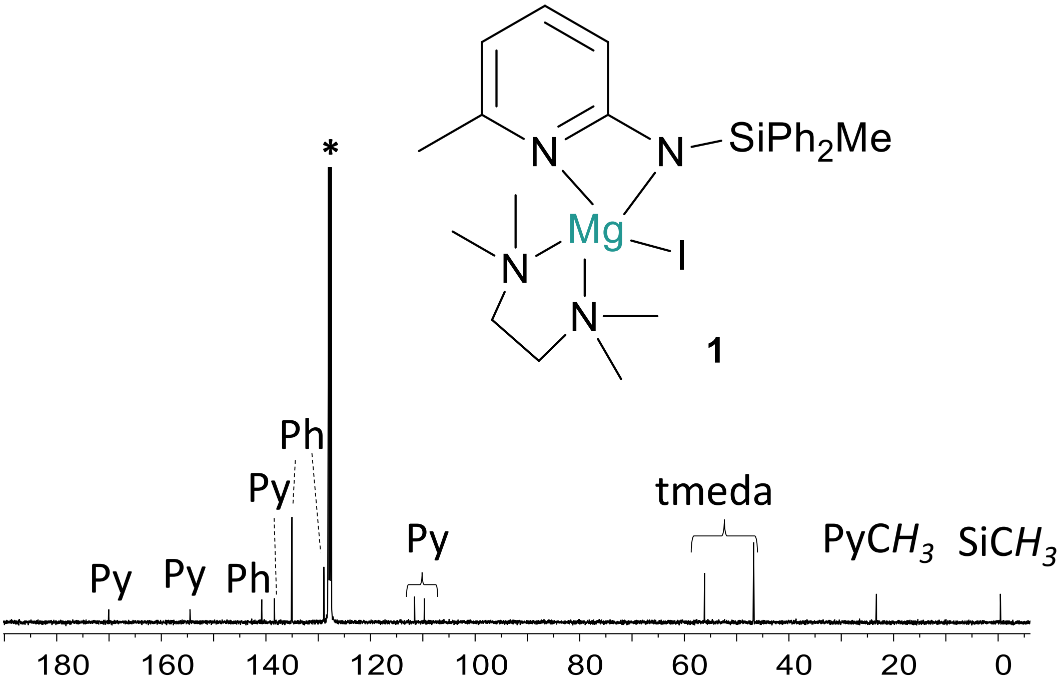

Figure S3: $^{13}C\{^{1}H\}$ NMR (101 MHz) spectra of **1** in C_6D_6 (*) at 25 °C.

S4.1.2 – Synthesis of 2 ($L^2MgI\cdot(tmeda)$)

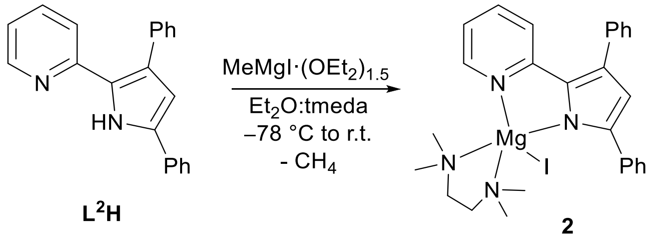

L²H **2**

Scheme S2: Synthesis of compound **2**

To a suspension of $MeMgI\cdot(Et_2O)_{1.5}$ (0.444 g, 1.6 mmol) in diethyl ether at −78 °C, a solution of **L^2H** (0.444 g, 1.5 mmol) in Et_2O (10 mL) was added dropwise over 1 h to afford a yellow suspension. The reaction was stirred for 4 h at −78 °C, and allowed to warm to −30 °C. TMEDA (0.9 mL, 6 mmol) was added, the resultant suspension stirred for a further 2 h, then allowed to warm to room temperature and volatiles removed *in vacuo*. Washing with cold hexanes afforded **2** as a green solid (0.771 g, 86%). Crystals of **2** of suitable quality for X-ray diffraction were grown from a saturated solution of the complex in diethyl ether at –30 °C.

^{1}H NMR (C_6D_6, 25 °C, 400 MHz): δ = 1.12 (t, *J* = 7.0 Hz, 3H, Et_2O), 1.70 (br s, 12H, *tmeda*) 1.90-2.34 (br s, 4H, *tmeda*), 3.26 (q, *J* = 6.9 Hz, 2H, Et_2O), 6.19 (ddd, *J* = 7.3, 5.3, 1.1 Hz, 1H, Py), 6.58 (ddd, *J* = 8.5, 7.2, 1.8 Hz, 1H, Py), 6.88 (s, 1 H, Pyr), 7.03 (tt, *J* = 7.4, 1.3 Hz, 1H, Ph^p), 7.19 (t, *J* = 6.9 Hz, 1H, Ph^p), 7.26 (t, *J* = 7.8 Hz, 2H, Ph^m), 7.31 (q, *J* = 8.6, 8.0 Hz, 2H, Ph^m), 7.68 (dd, *J* = 8.1 Hz, 1.2 Hz, 2H, Ph^o), 8.05 (dd, *J* = 8.2 Hz, 1.2 Hz, 2H, Ph^o), 8.62 (ddd, *J* = 5.4, 1.9, 0.9 Hz, 1H, Py).

^{13}C{^{1}H} NMR (C_6D_6, 25 °C, 101 MHz): δ = 15.3 (Et_2O), 46.5 (tmeda), 47.2 (tmeda), 65.7 (Et_2O), 114.6 (Ar-*C*H), 117.3 (Ar-*C*H), 118.4 (Ar-*C*H), 126.0 (Ar-*C*H), 126.1 (Ar-*C*H), 126.5 (Ar-*C*H), 128.6 (Ar-*C*H), 129.2 (Ar-*C*H), 129.5 (Ar-*C*H), 131.8 (Ar-*C*), 134.0 (Ar-*C*), 137.6 (Ar-*CH*), 139.2 (Ar-*C*), 139.9 (Ar-*C*), 146.4 (Ar-*C*), 147.7 (*C*H), 155.9 (Ar-*C*).

$C_{27}H_{31}IMgN_4\cdot(C_{10}H_4O)_{0.5}$ (599.18): calc'd (%) C 58.07, H 6.05, N 9.34; found (%) C 57.93, H 5.95, N 9.26.

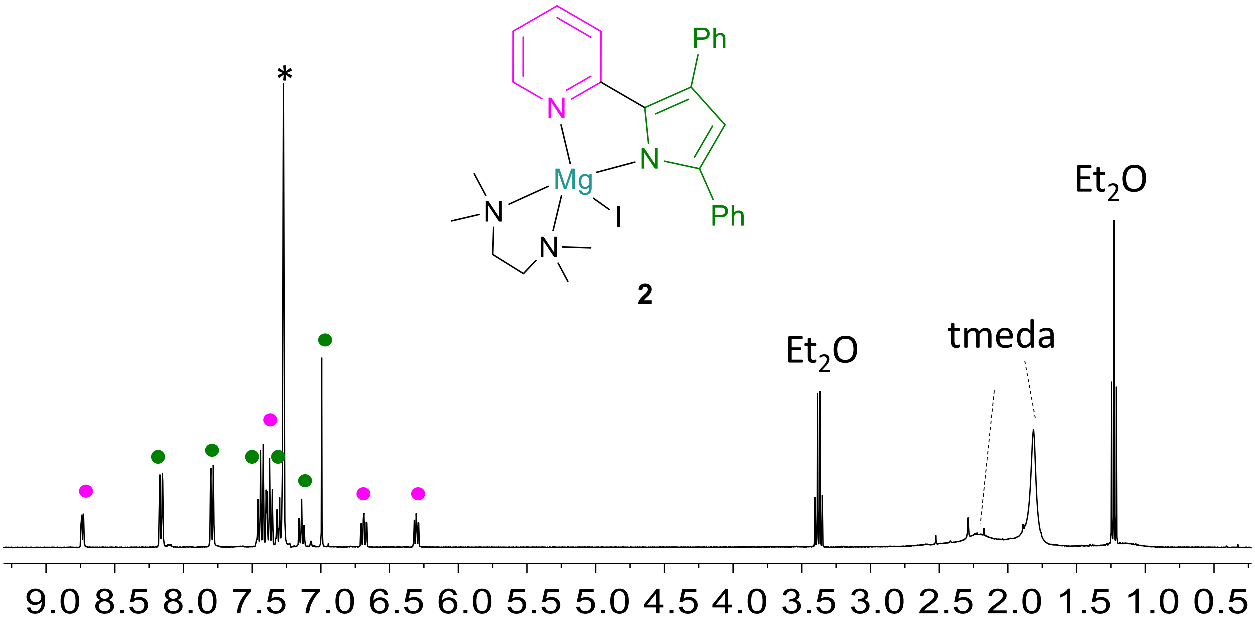

Figure S4: ^{1}H NMR (400 MHz) spectra of **2** in C_6D_6 (*) at 25 °C.

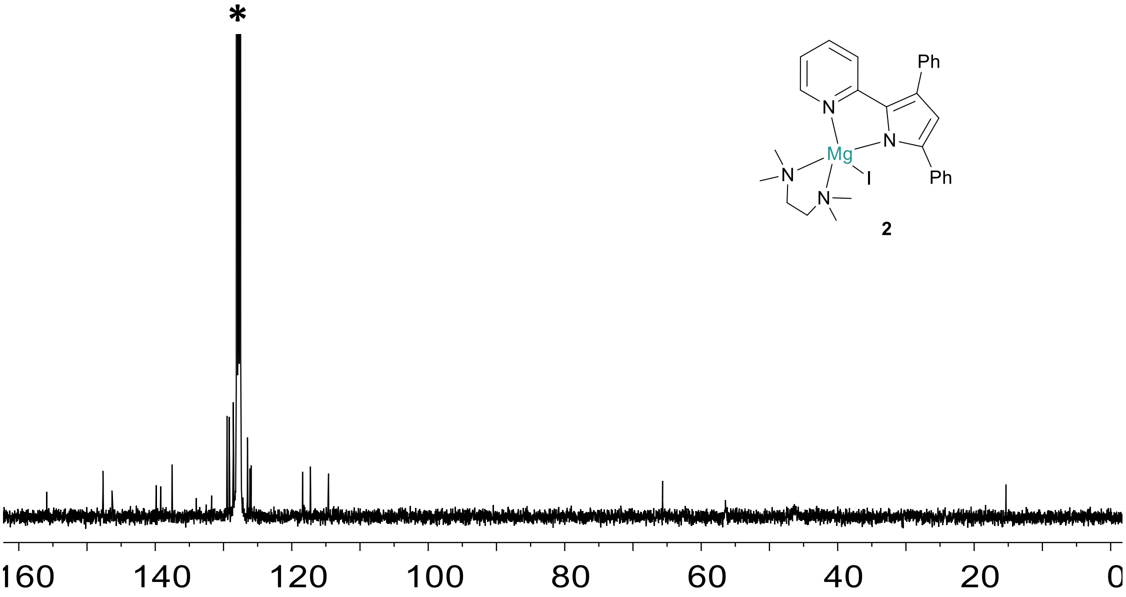

Figure S5: $^{13}C\{^{1}H\}$ NMR (101 MHz) spectra of **2** in C_6D_6 (*) at 25 °C.

S4.1.3 – Synthesis of $Me_2ND{\cdot}BH_3$

Synthesis adapted from a literature procedure.[12] $Me_2NH{\cdot}BH_3$ (500 mg, 8.49 mmol) was dissolved in D_2O (2 mL) and stirred for 2 h at room temperature. The solution was washed with DCM (3 x 10 mL) and the organic layer was separated and dried over Na_2CO_3. Removal of volatiles *in vacuo* afforded $Me_2ND{\cdot}BD_3$ as a white crystalline solid (303.8 mg, 5.07 mmol, 60%). No NH signal could be detected by ^1H NMR spectroscopy.

^1H NMR (C_6D_6, 25 °C, 400 MHz): δ = 1.75 (s, 6H, CH_3), 1.78–2.58 (br q, *J* = 97 Hz, 3H, BH_3).

^{11}B NMR (C_6D_6, 25 °C, 96 MHz): δ = −13.0 (q, *J* = 97 Hz, BH_3).

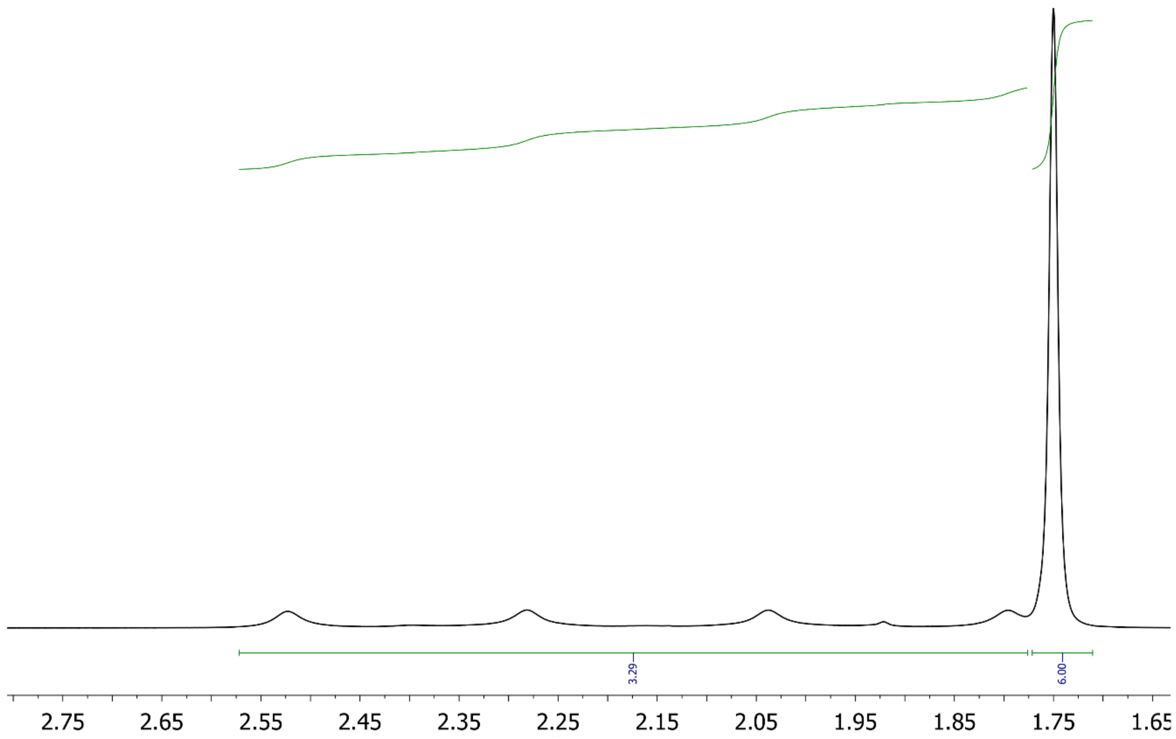

Figure S6: ^1H NMR (400 MHz) spectra of $Me_2ND{\cdot}BH_3$ in C_6D_6 at 25 °C.

S4.1.4 – Synthesis of $Me_2NH{\cdot}BD_3$

We attempted to synthesise $Me_2NH{\cdot}BD_3$ *via* a literature procedure, wherein $Me_2NH{\cdot}HCl$ was reacted with $NaBD_4$ and purified by aqueous work up.[13] Contrary to the published report, this does not afford pure $Me_2NH{\cdot}BD_3$, but a mixture of $Me_2NH{\cdot}BD_3$ and $Me_2NH{\cdot}BH_3$, which is likely due to H/D exchange with the water used in the aqueous work-up. These compounds display two distinct signals for the Me groups in the ^1H NMR spectrum (Figure S7), and were originally assigned as a doublet in the literature.[13] Furthermore, we observe two distinct signals in the ^{13}C NMR spectrum in C_6D_6 solution (Figure S8). The product was also characterised by IR spectroscopy, which revealed clear B–H stretches. Characterisation data for the products of this reaction is given below.

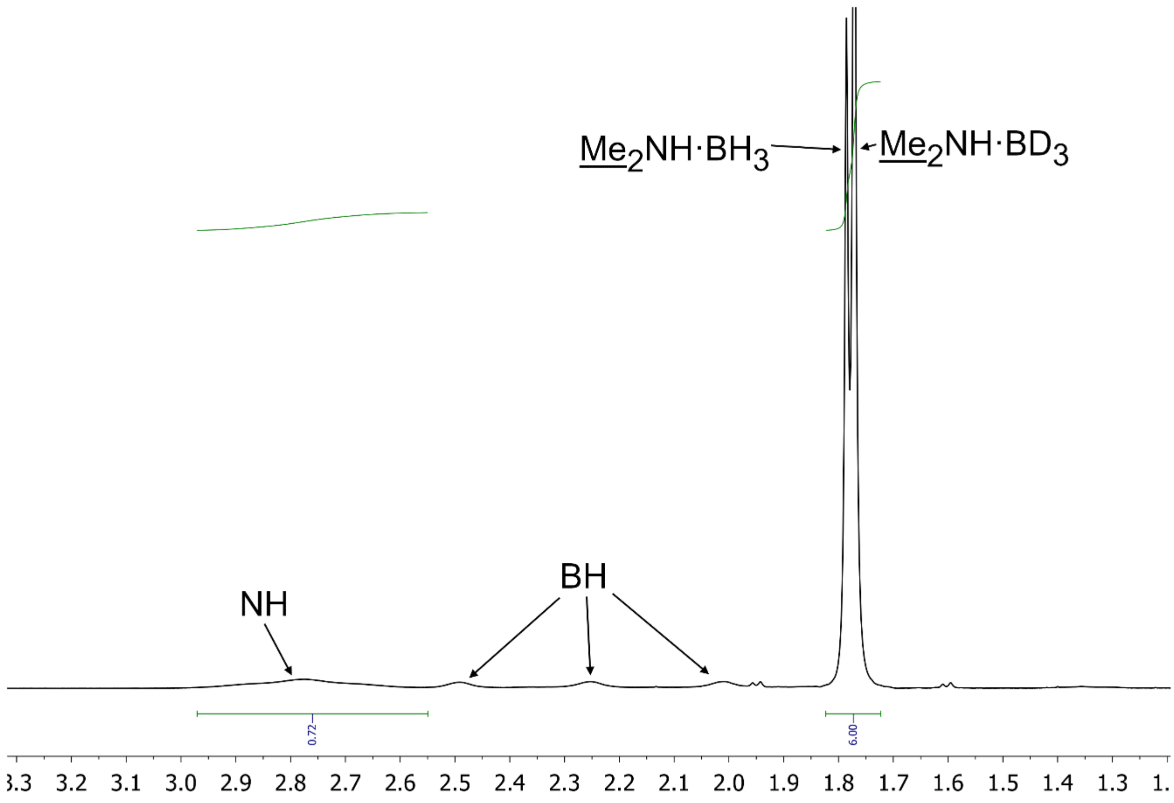

Figure S7: ^{1}H NMR (400 MHz) spectra of mixed sample of $Me_2NH{\cdot}BD_3$ and $Me_2ND{\cdot}BH_3$ in C_6D_6 at 25 °C.

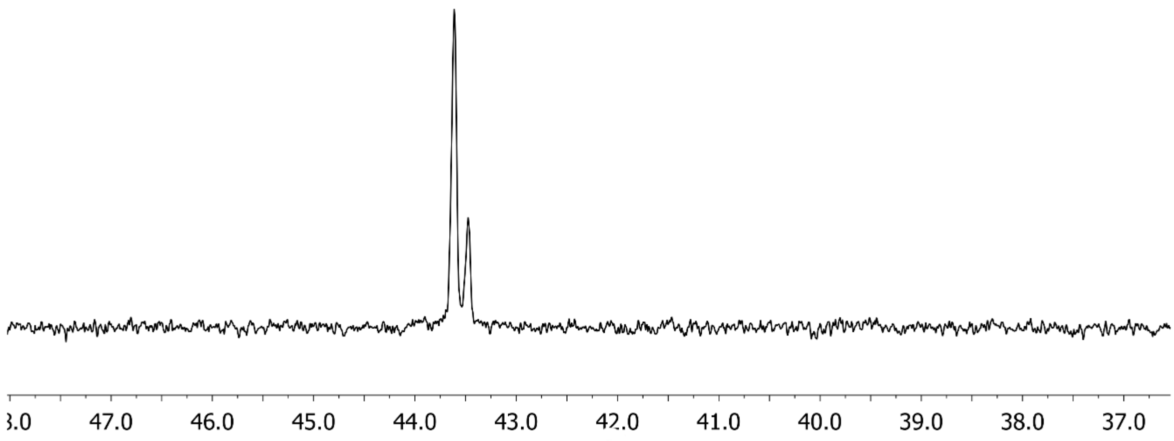

Figure S8: $^{13}C\{^{1}H\}$ NMR (101 MHz) spectra of mixed sample of $Me_2NH{\cdot}BD_3$ and $Me_2ND{\cdot}BH_3$ in C_6D_6 at 25 °C.

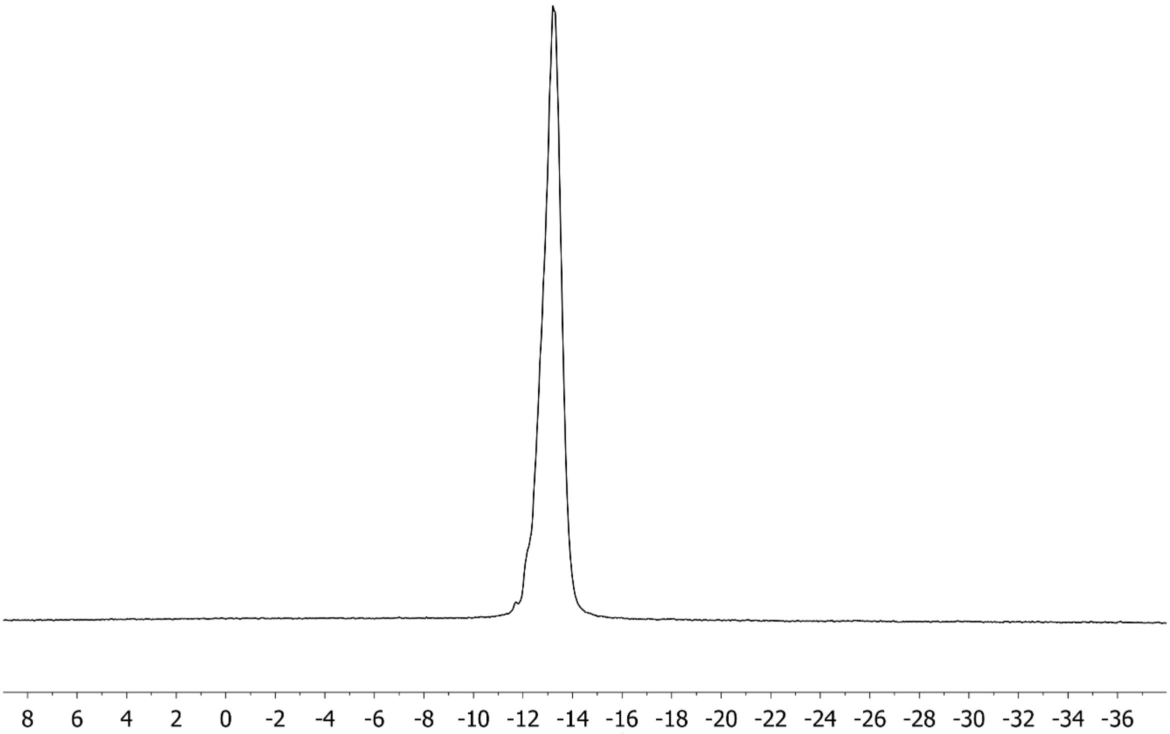

Figure S9: ^{11}B NMR (96 MHz) spectra of mixed sample of $Me_2NH{\cdot}BD_3$ and $Me_2ND{\cdot}BH_3$ in C_6D_6 at 25 °C.

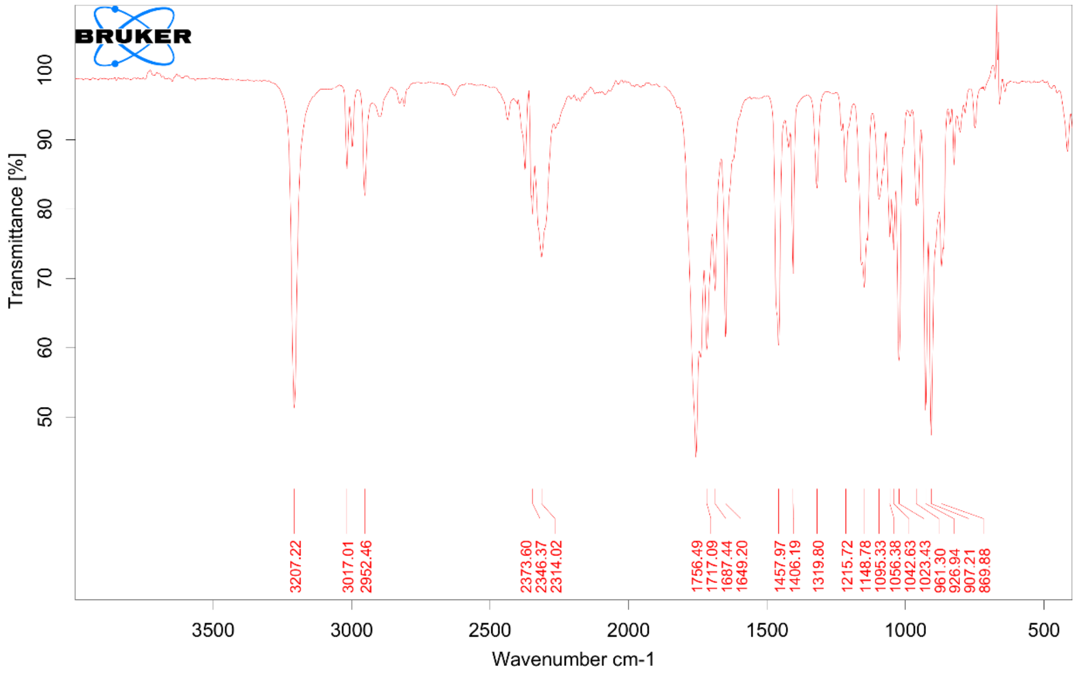

Figure S10: IR spectrum (ATR) of mixed sample of $Me_2NH{\cdot}BD_3$ and $Me_2ND{\cdot}BH_3$. υ(B–H) = 2374, 2346, 2314 cm^{-1}; υ(B–D) = 1756, 1717, 1687 cm^{-1}.

An alternative synthetic route was attempted, in which the reaction was carried out and worked up under completely anhydrous conditions (see below). This gave a higher degree of deuteration, but the product still contained significant amounts (*ca.* 20%) of BH_3 containing product (Figure S11). This seems to be the result of deuterium exchange with $Me_2NH{\cdot}HCl$, as ^1H NMR and IR spectroscopy of the product indicated a loss of proton label at N and N–D stretching bands (Figure S14).

Anhydrous Procedure: To a Schlenk flask charged with $Me_2NH{\cdot}HCl$ (612 mg, 7.5 mmol), $NaBD_4$ (330 mg, 7.9 mmol), and a stirrer bar; pre-cooled (0 °C) THF (5 mL) was added and the resultant slurry stirred at 0 °C for 1 h. The reaction was then allowed to warm to room temperature and stirred overnight. Volatiles were removed *in vacuo* and the product was isolated by vacuum sublimation as a white crystalline solid (228 mg). Characterisation data for this product is shown below.

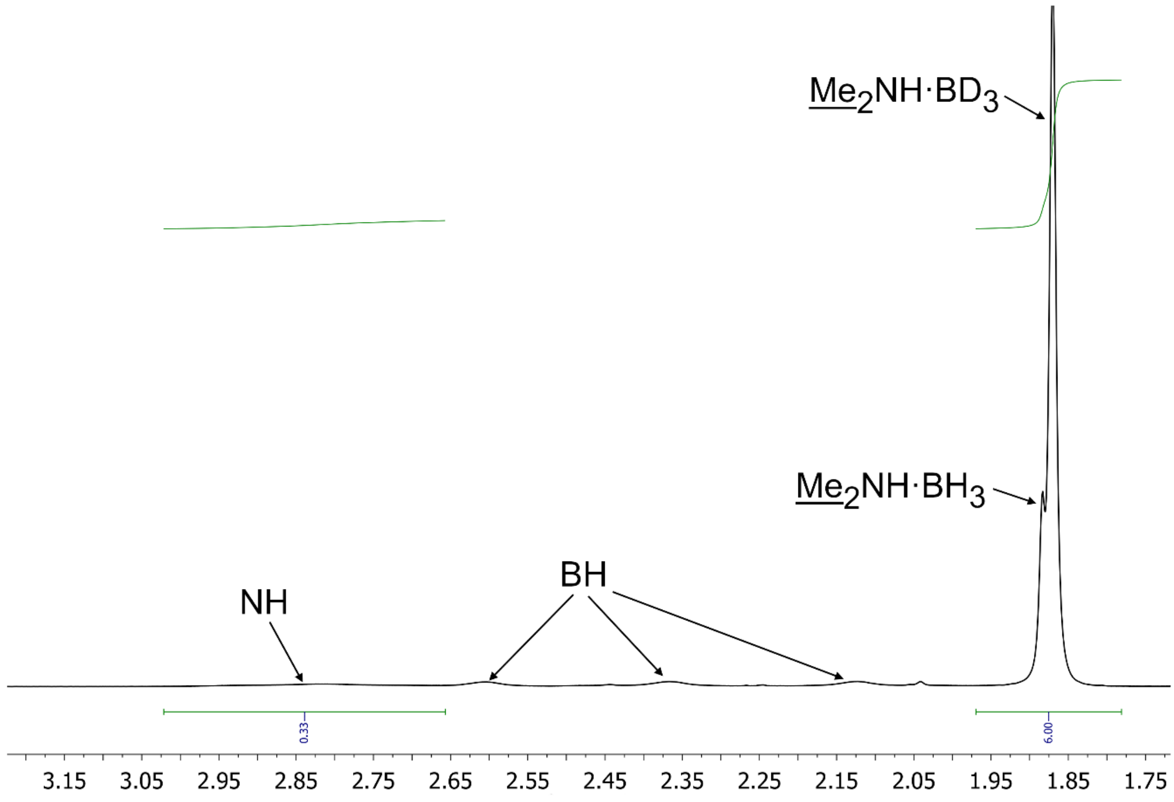

Figure S11: ^1H NMR (400 MHz) spectra of anhydrously prepared sample of $Me_2NH{\cdot}BD_3$ and $Me_2ND{\cdot}BH_3$ in C_6D_6 at 25 °C.

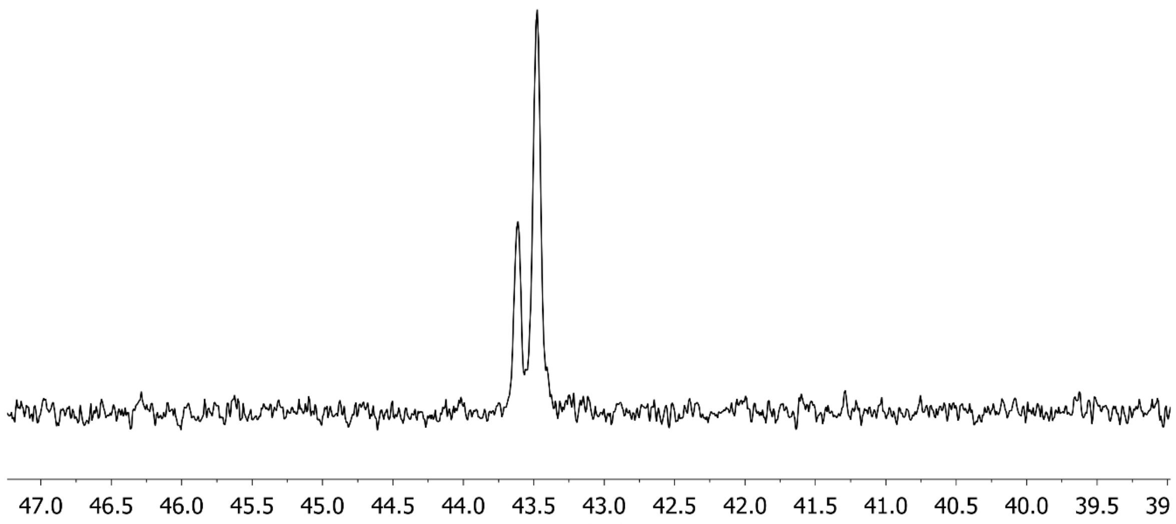

Figure S12: $^{13}C\{^{1}H\}$ NMR (101 MHz) spectra of anhydrously prepared sample of $Me_2NH{\cdot}BD_3$ and $Me_2ND{\cdot}BH_3$ in C_6D_6 at 25 °C.

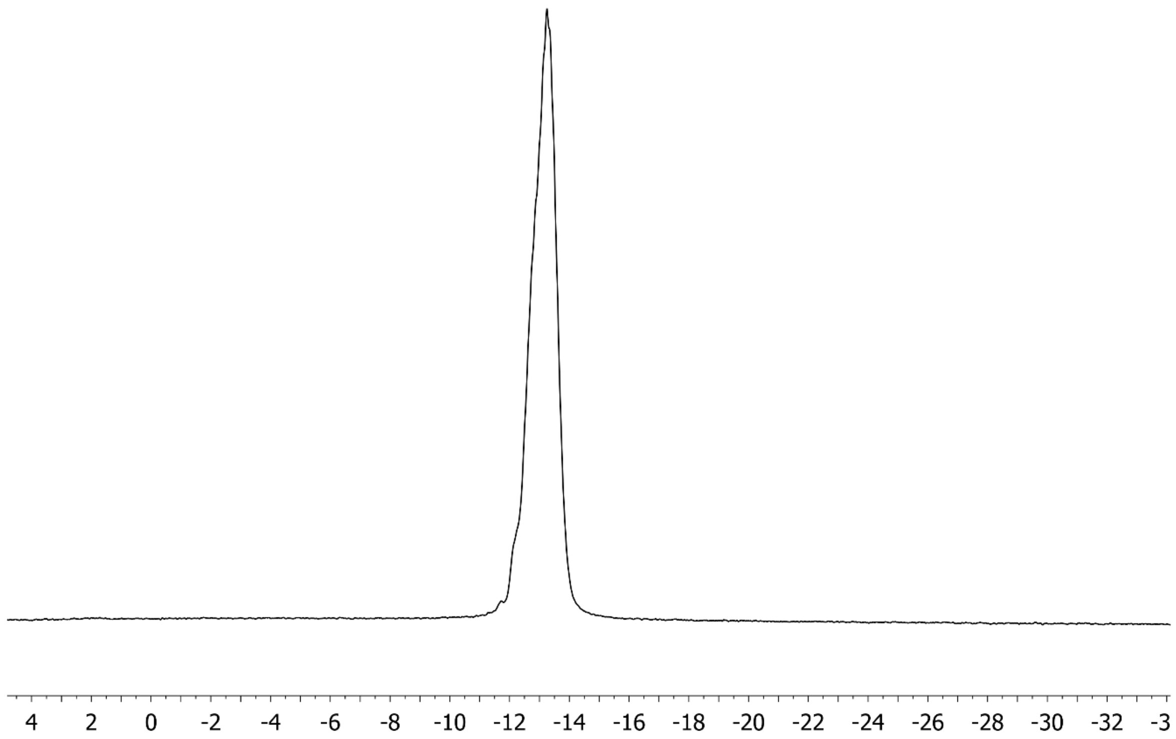

Figure S13: ^{11}B NMR (96 MHz) spectra of anhydrously prepared sample of $Me_2NH{\cdot}BD_3$ and $Me_2ND{\cdot}BH_3$ in C_6D_6 at 25 °C.

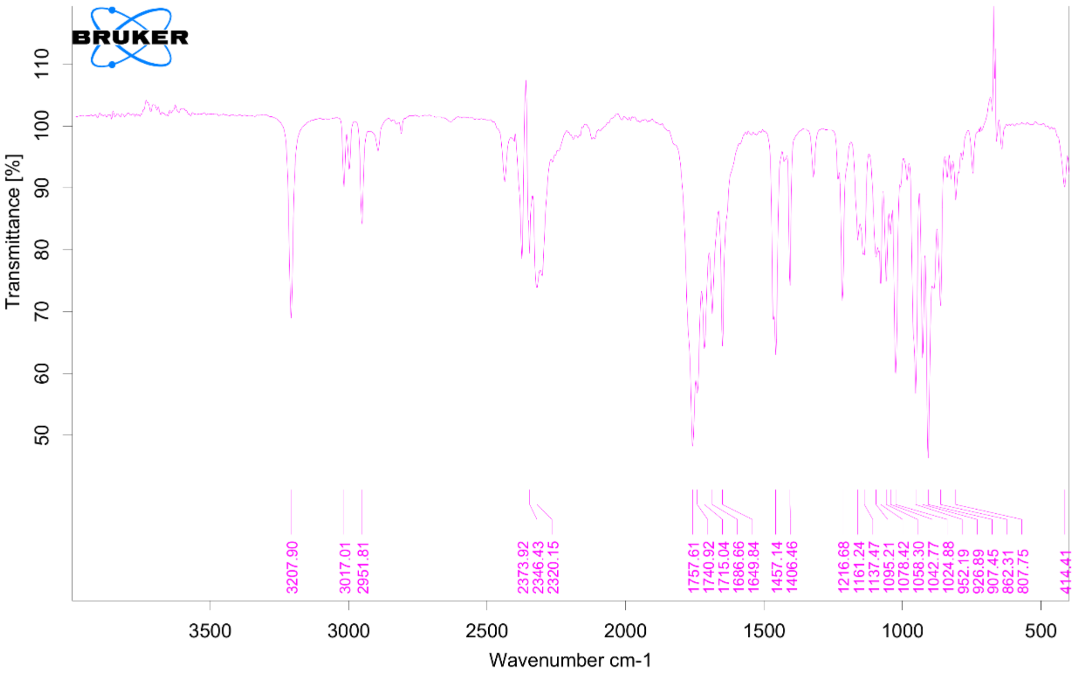

Figure S14: IR spectrum (ATR) of mixed sample of $Me_2NH{\cdot}BD_3$ and $Me_2ND{\cdot}BH_3$.

S4.2 – Reaction Monitoring

S4.2.1 – Standard procedure for the catalytic dehydrocoupling of $Me_2NH{\cdot}BH_3$

In a Young's NMR tube 1 mol% (0.0017 mmol), 5 mol% (0.0085 mmol) or 10 mol% (0.0170 mmol) of catalyst **1** or **2** and $Me_2NH{\cdot}BH_3$ (10 mg, 0.170 mmol) were dissolved in 0.6 mL of the corresponding solvent (C_6D_6 or THF). The reaction was heated to 60 °C in an oil bath and progress was monitored by ^{1}H and/or ^{11}B NMR spectroscopy at predetermined time-points. In the case of THF, a capillary of C_6D_6 was also added to the NMR tube to provide a lock.

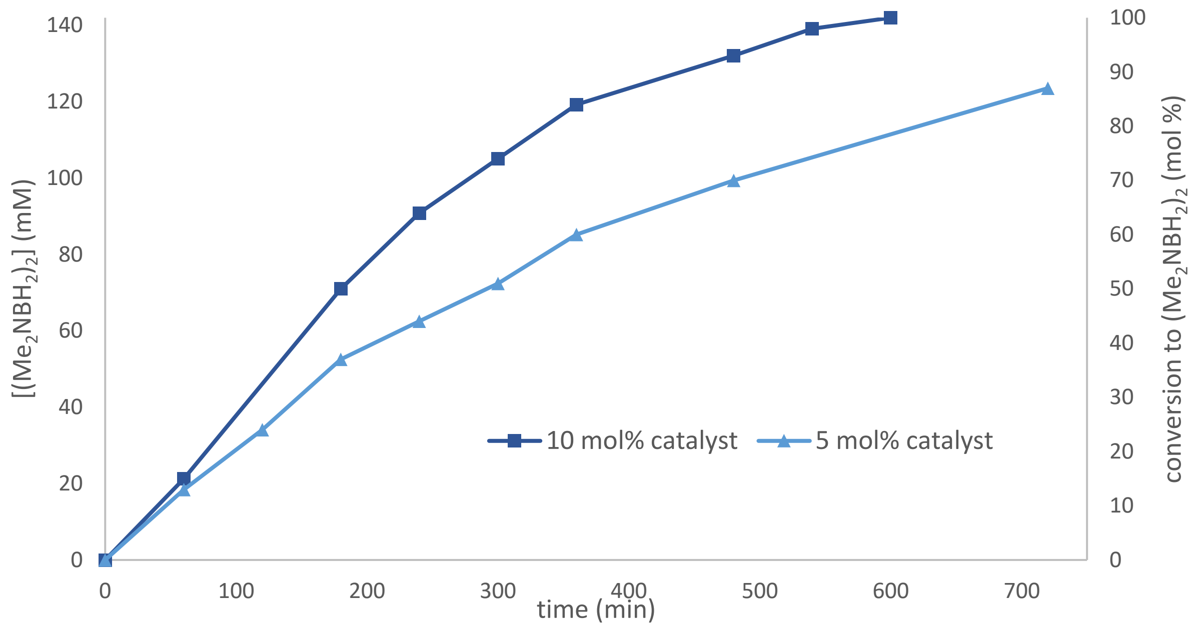

Figure S15: Product concentration (mM) and conversion (mol%) vs. time (min) for the dehydrocoupling of $Me_2NH{\cdot}BH_3$ with 5 mol% or 10 mol% of **1** in C_6D_6 at 60 °C, as determined by discontinuous NMR measurements.

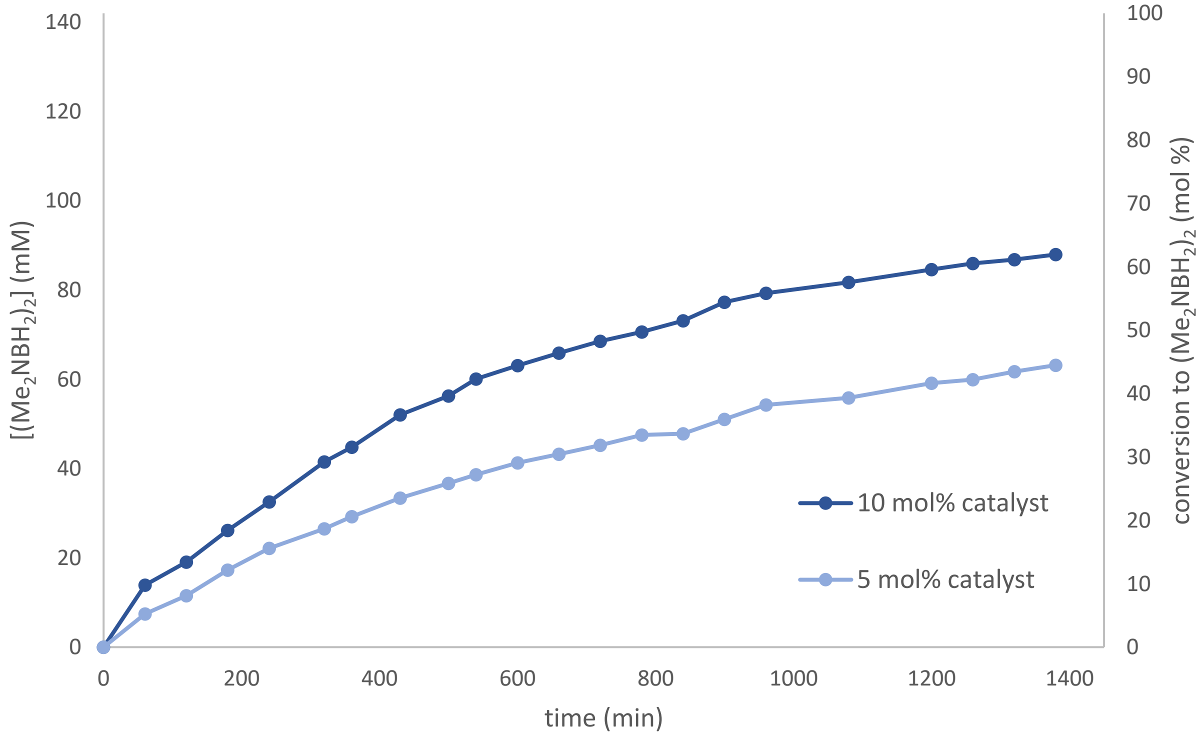

Figure S16: Product concentration (mM) and conversion (mol%) vs. time (min) for the dehydrocoupling of $Me_2NH{\cdot}BH_3$ with 5 mol% or 10 mol% of **1** in THF at 60 °C, as determined by discontinuous NMR measurements.

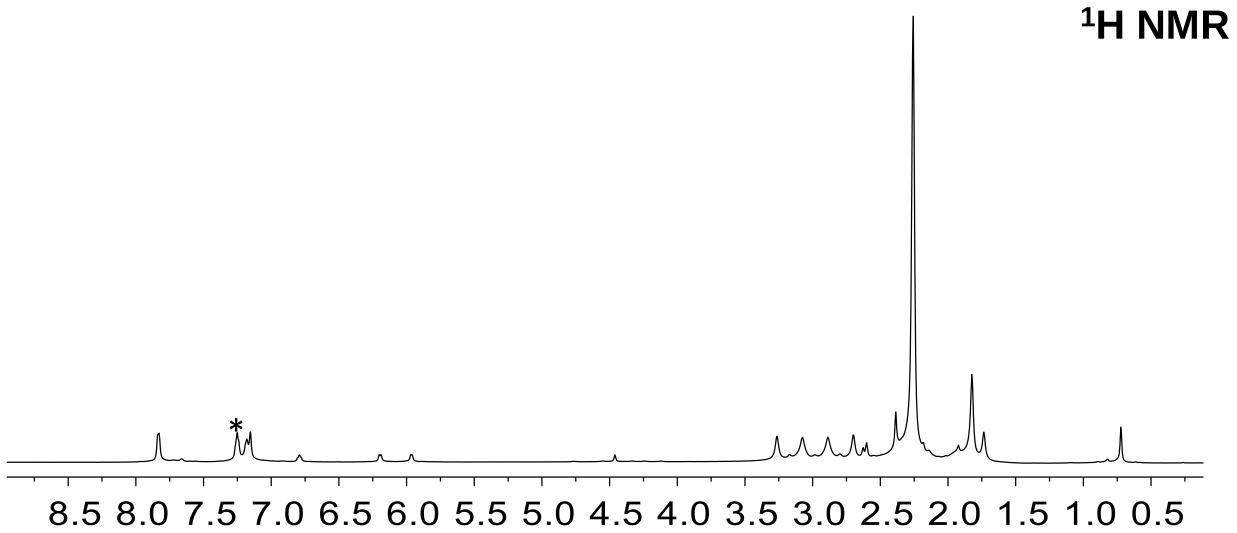

Figure S17: ^{1}H NMR (400 MHz) spectrum for the dehydrocoupling of $Me_2HN·BH_3$ with 10 mol% **1** in C_6D_6 (*) after 1.5 h at 60 °C.

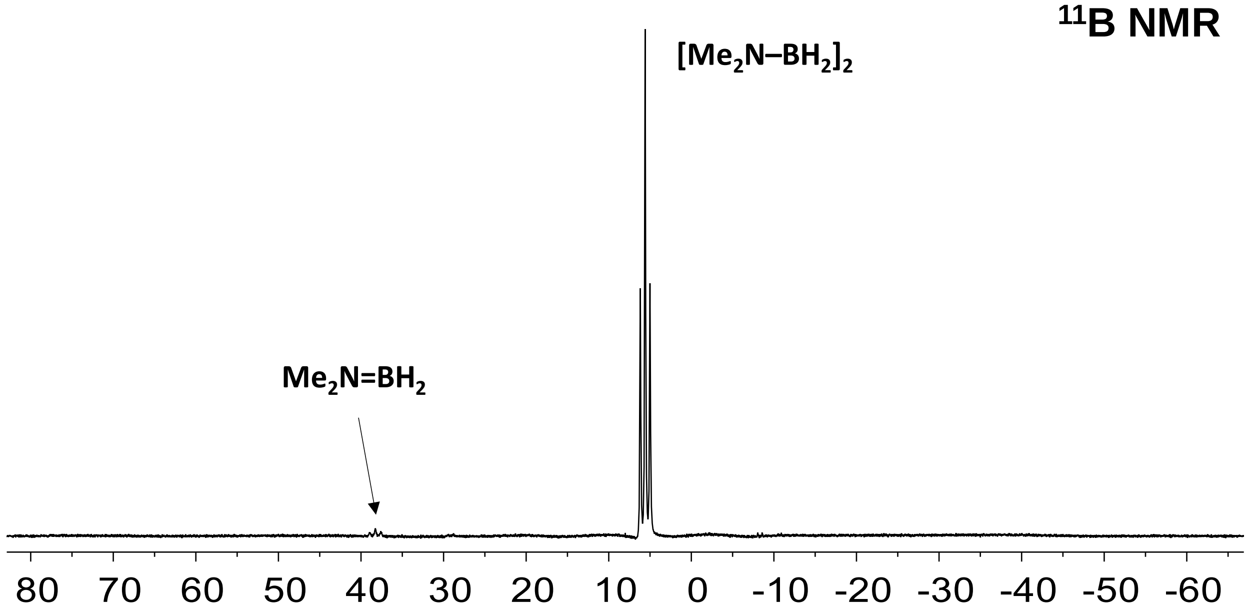

Figure S18: ^{11}B NMR (96 MHz) spectrum for the dehydrocoupling of $Me_2HN·BH_3$ with 10 mol% **1** in C_6D_6 after 1.5 h at 60 °C.

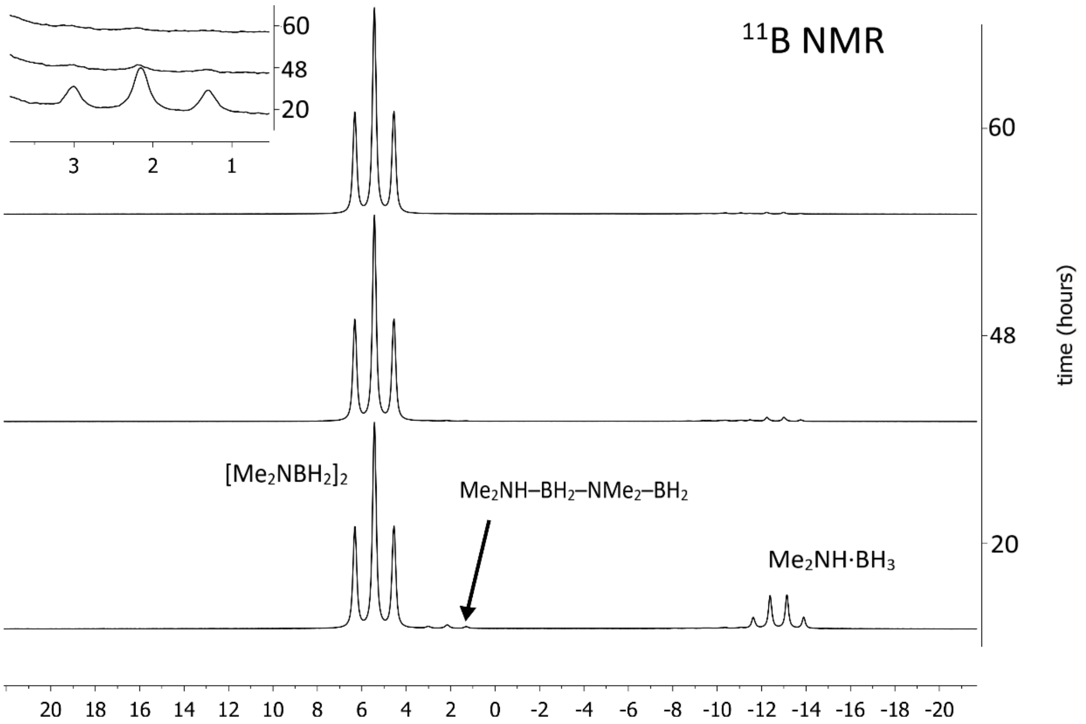

Figure S19: Stacked ^{11}B NMR (96 MHz) spectra for the dehydrocoupling of $Me_2NH{\cdot}BH_3$ with 1 mol% **1** in C_6D_6 after 20, 48, and 60 h at 60 °C. Inset shows spectral zoom of signals from linear intermediate **5**.

S4.2.2 – Qualitative *in situ* monitoring by ^{1}H NMR spectroscopy

In a Young's NMR tube 5 mol% (0.0085 mmol) of catalyst **1** and $Me_2NH{\cdot}BH_3$ (10 mg, 0.170 mmol) were dissolved in 0.6 mL of C_6D_6. An initial ^{1}H NMR spectrum was recorded at 25 °C (Figure S20a), revealing no reaction had occurred. The temperature in the spectrometer was increased to 60 °C, and ^{1}H NMR spectra were recorded at regular intervals (Figure S20b). Once no starting material was observable by ^{1}H NMR spectroscopy, the temperature was reduced to 25 °C, and a final ^{1}H NMR spectrum was recorded (Figure S20c).

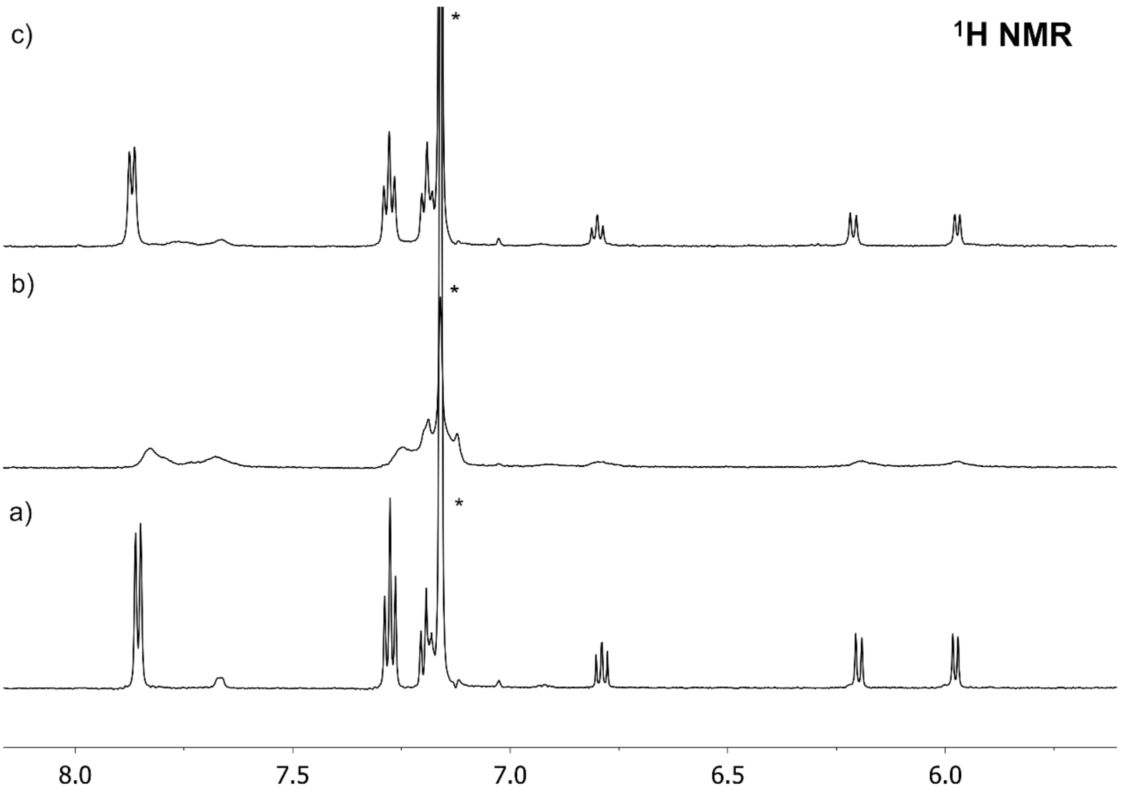

Figure S20: Aromatic region of the ^{1}H NMR (600 MHz) spectrum of the catalytic dehydrocoupling of $Me_2NH{\cdot}BH_3$ with 5 mol% catalyst in C_6D_6 (*). Spectra recorded at (a) 0% conversion, 25 °C (b) *ca.* 50% conversion, 60 °C (c) 100% conversion, 25 °C. No appreciable change in signals from the catalyst is observed between spectra (a) and (c).

S4.2.3 – Standard procedure for the catalytic dehydrocoupling of *i*$Pr_2NH{\cdot}BH_3$

In a Young's NMR tube 5 mol% (0.0088 mmol) of catalyst **1** and *i*$Pr_2NH{\cdot}BH_3$ (20 mg, 0.175 mmol) were dissolved in 0.6 mL of C_6D_6. The reaction was either left at room temperature or heated to 60 °C in an oil bath and progress was monitored by ^1H and ^{11}B NMR spectroscopy at predetermined time-points. The product (*i*$Pr_2N{=}BH_2$) was identified by comparison of ^1H and ^{11}B NMR spectra with literature data.[13]

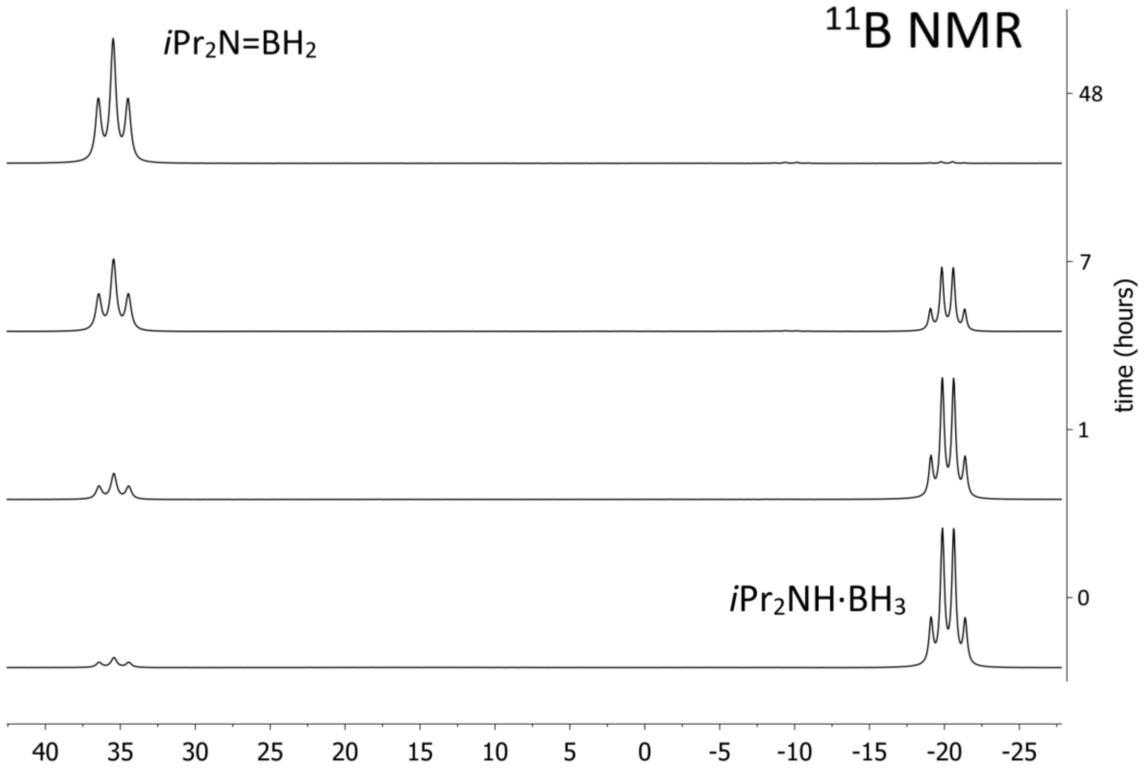

Figure S21: Stacked ^{11}B NMR (96 MHz) spectra showing conversion of *i*$Pr_2NH{\cdot}BH_3$ to *i*$Pr_2N{=}BH_2$ at room temperature in the presence of 5 mol% **1**.

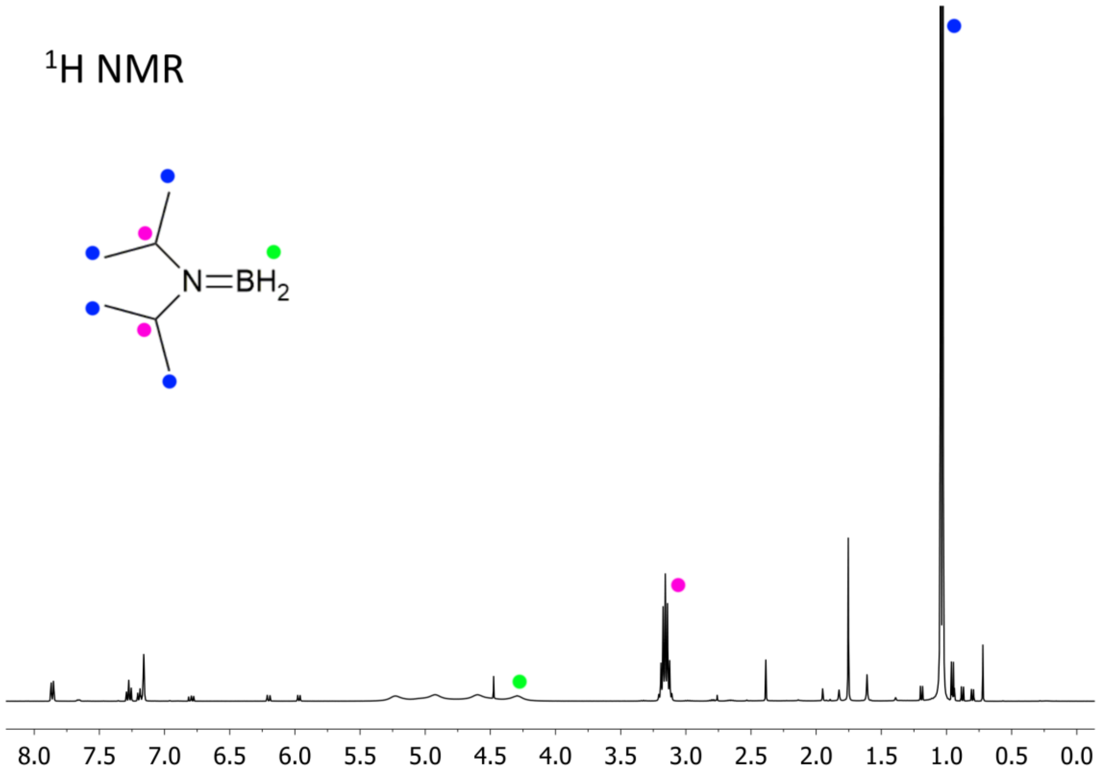

Figure S22: ^{1}H NMR (400 MHz) spectrum for the dehydrocoupling of *i*$Pr_2HN{\cdot}BH_3$ with 5 mol% **1** in C_6D_6 after 48 h at room temperature

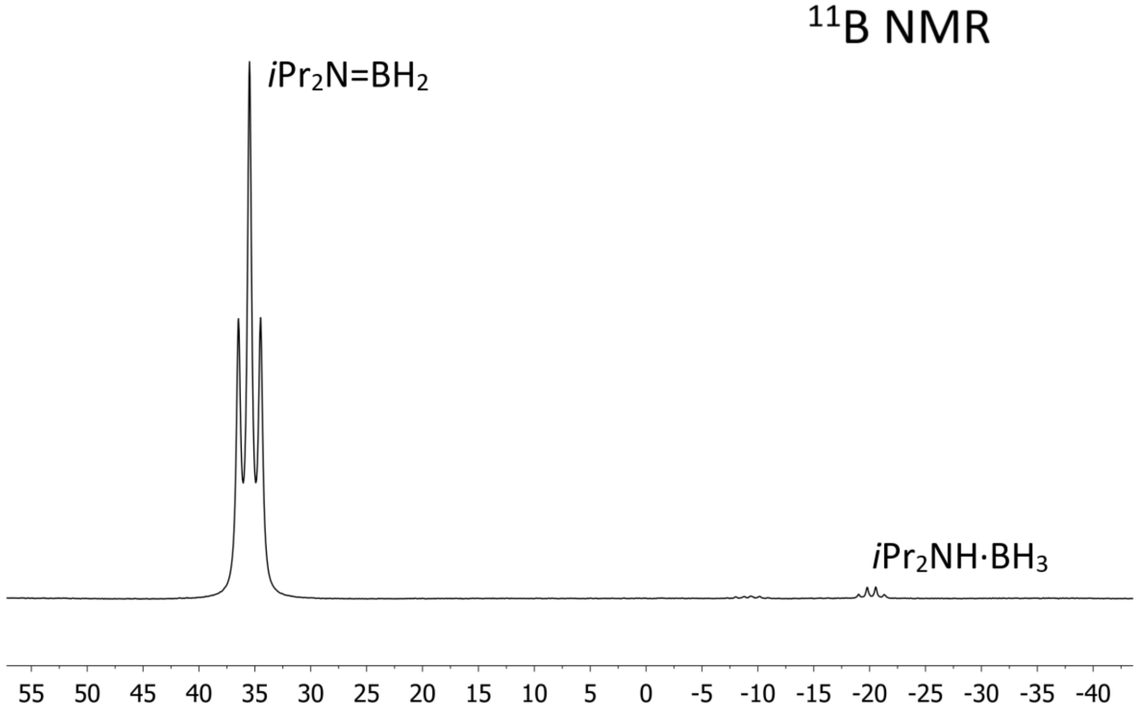

Figure S23: ^{11}B NMR (96 MHz) spectrum for the dehydrogenation of *i*$Pr_2HN{\cdot}BH_3$ with 5 mol% **1** in C_6D_6 after 1 h at 60 °C

S4.2.4 – Hydrogen Evolution Experiments

Catalytic dehydrocoupling of $Me_2NH{\cdot}BH_3$: A solution of 10 mol% (96.3 mg, 0.166 mmol), 5 mol% (48.2 mg, 0.083 mmol) or 2 mol% (19.3 mg, 0.033 mmol) of catalyst **1** in 5 mL of toluene, stirred at 200 rpm, was heated to 60 °C until equilibrium vapour pressure was reached (*i.e.* no change in volume was observed for 15 min, typically after 45 min). Subsequently, a solution of $Me_2NH{\cdot}BH_3$ (98.2 mg, 1.66 mmol) in 5 mL of toluene was injected, and reaction progress monitored by gas evolution.

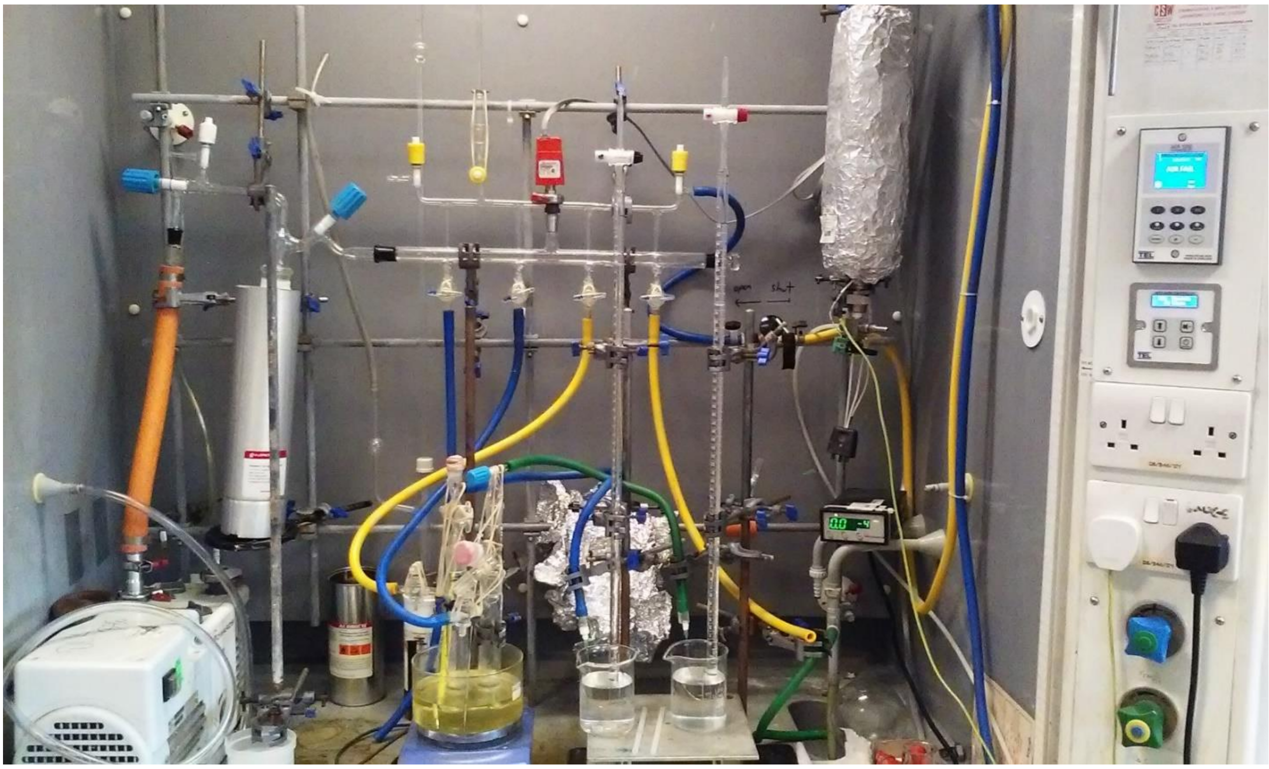

Figure S24: Set-up for the measurement of H_2 formation in an open system.

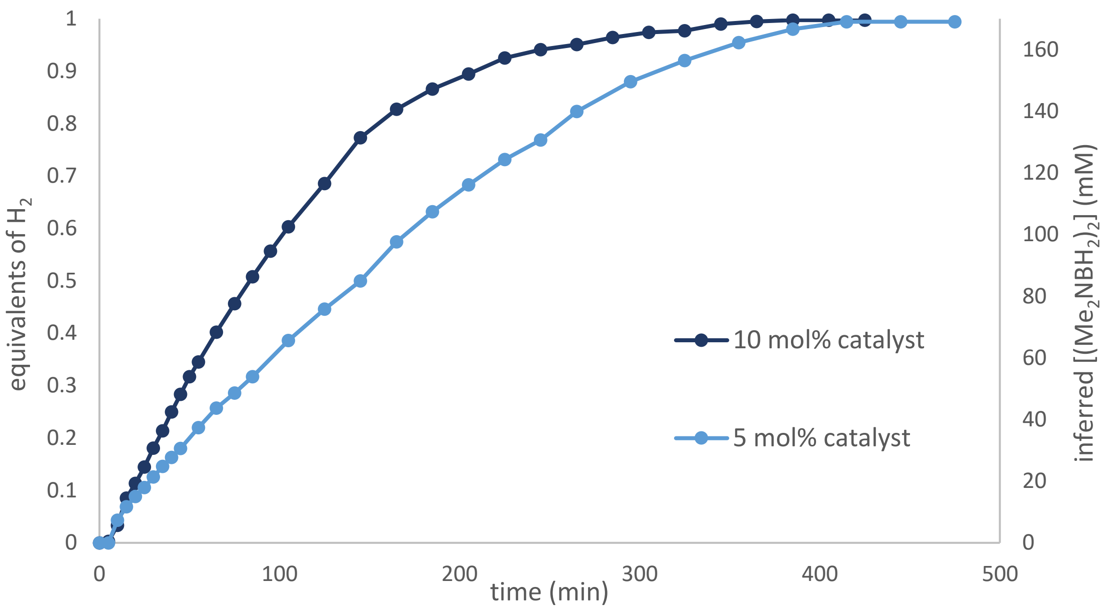

Figure S25: H_2 equivalents (mol H_2) and inferred product concentration (mM) vs. time (min) for the dehydrocoupling of $Me_2NH{\cdot}BH_3$ using **1** with 5 mol% and 10 mol% in toluene at 60 °C. Data obtained by volumetric measurements of hydrogen evolution.

Recyclability experiments: The above procedure was carried out for 10 mol% of **1** (96.3 mg, 0.166 mmol). After completion, the connection to the measuring burette was shut, and the Schlenk tube kept open to argon at 60 °C overnight to ensure complete consumption of any remaining starting material. The argon was then disconnected, the pressure left to equilibrate, and a further equivalent of $Me_2NH{\cdot}BH_3$ (98.2 mg, 1.66 mmol) in 2 mL of toluene injected. The procedure was repeated for a third time, with prior volume reduction *in vacuo* to *ca.* 8 mL.

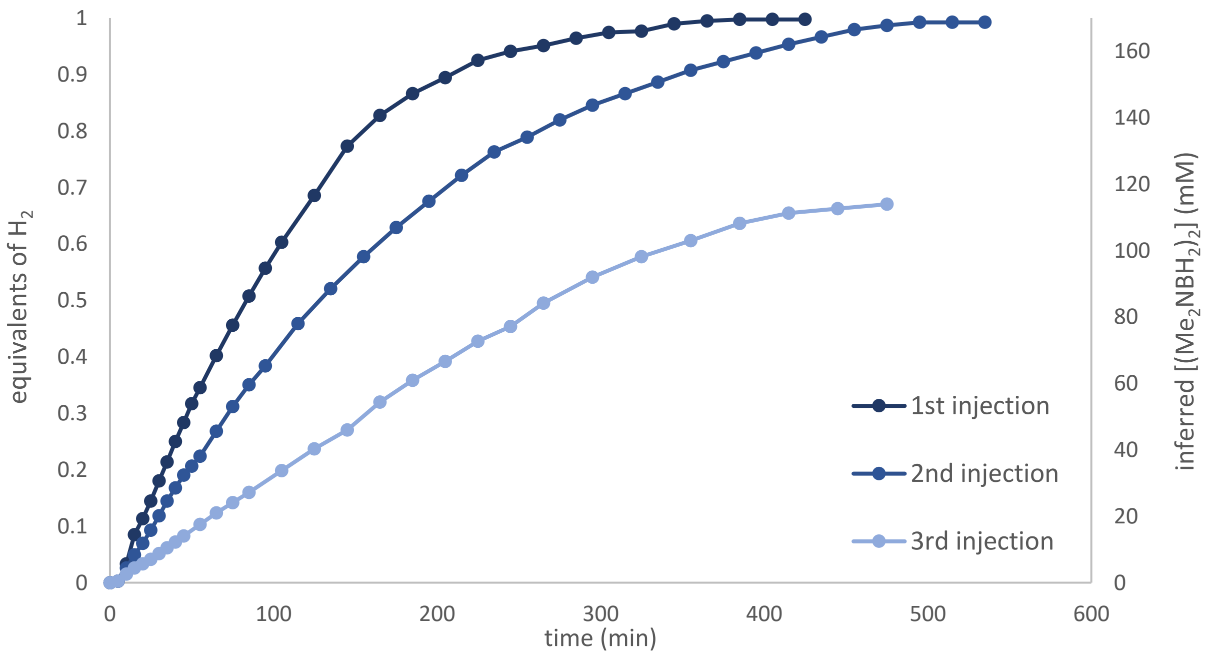

Figure S26: H_2 equivalents (molH_2) and inferred product concentration (mM) vs. time (min) for the dehydrogenation of $Me_2NH{\cdot}BH_3$ using **1** (10 mol%) in toluene at 60 °C after three cycles using the same catalyst.

S4.3 – Kinetic Experiments

S4.3.1 – Procedure for determining reaction orders

In a Young's NMR tube 0.6 mL of a stock solution (A, B, or C) containing $Me_2NH{\cdot}BH_3$ and catalyst **1** in C_6D_6 (A: 0.29 M $Me_2NH{\cdot}BH_3$, 28 mM **1**; B: 0.29 M $Me_2NH{\cdot}BH_3$, 22 mM **1**; C: 0.29 M $Me_2NH{\cdot}BH_3$, 15 mM **1**) was prepared and frozen at −78 °C to prevent reaction initiation. The sample was transferred to a Bruker AV(III) 600 spectrometer, and a ^{11}B NMR spectra recorded at 15 °C, which showed no reaction. The temperature was then rapidly ramped to 60 °C. The temperature was monitored using a thermocouple located within the probe. Once the temperature stabilised sufficiently to allow locking to the C_6D_6, ^{11}B NMR spectra were recorded at regular intervals until at least 95% of the starting material had been consumed (16 scans, 5 second pulse delay, 4 second delay between experiments. T1 values: $Me_2NH{\cdot}BH_3$ 46.9 ms; $[Me_2NBH_2]_2$ 90.1 ms). Conversion was quantified by integration of the ^{11}B NMR spectra, and absolute concentrations calculated from the known initial concentration of $Me_2NH{\cdot}BH_3$ (290 mM). Reaction rate (υ, mM s^{-1}) as a function of time was determined by fitting the data (concentration of $Me_2NH{\cdot}BH_3$ vs time) to a 4th order polynomial curve, then differentiating with a Savitzky–Golay smoothing algorithm, polynomial order 2. This was then used to determine Reaction rate (υ) as a function of substrate concentration ($[Me_2NH{\cdot}BH_3]$, mM)

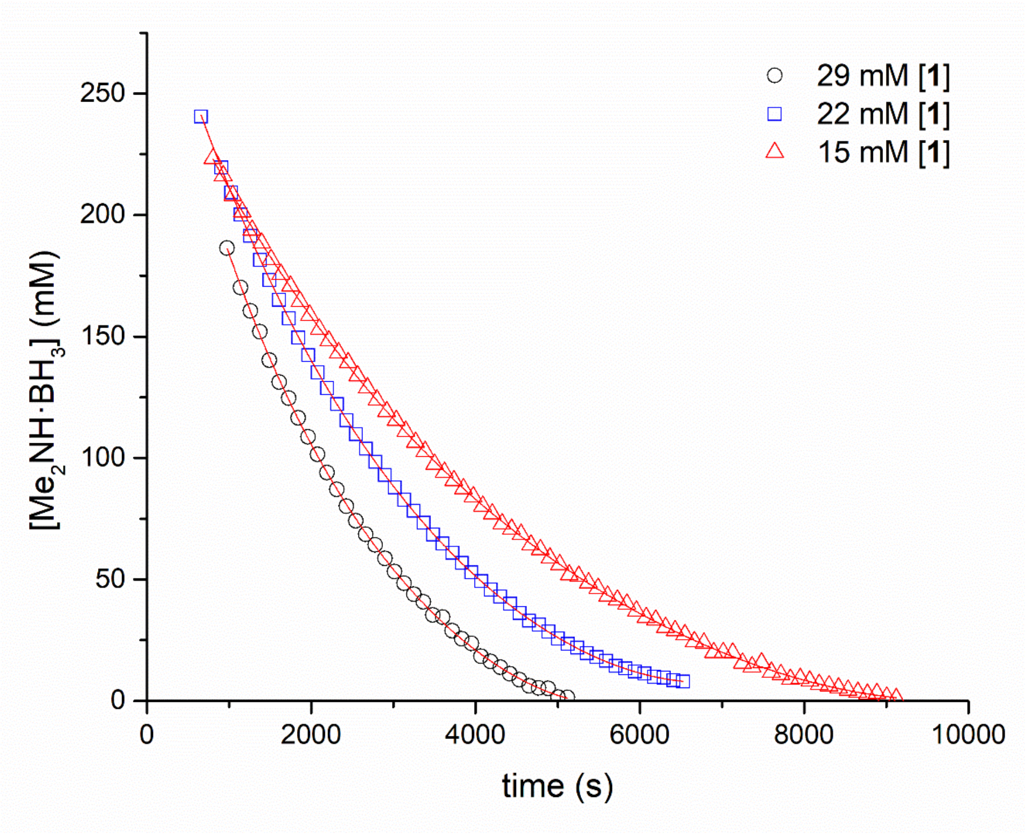

Figure S27: Concentration of $Me_2NH{\cdot}BH_3$ (mM) vs time (s) for reactions at 3 different concentrations of catalyst **1** (29 mM, 22 mM, 15 mM). All reactions carried out at 60 °C in C_6D_6 with an initial substrate concentration of 0.29 M. Polynomial fits of concentration data shown as red lines. All time values are given relative to an approximate start time, which is the time at which temperature ramping to 60 °C was begun.

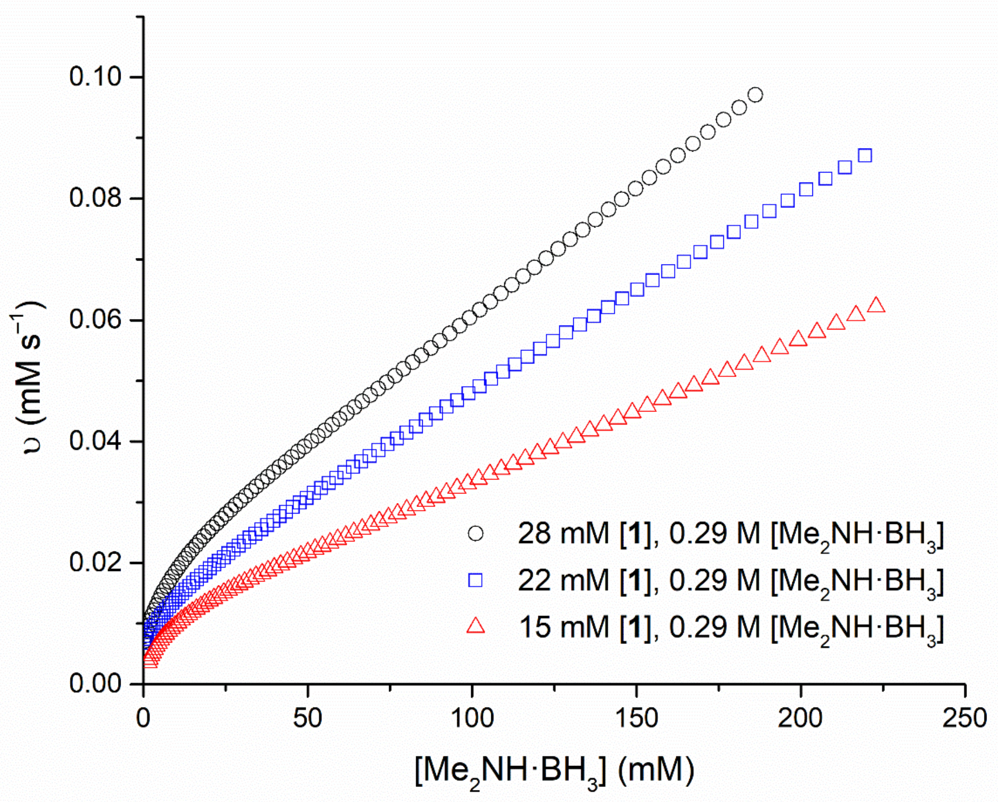

Figure S28: Plot of rate (υ, mM s^{-1}) vs concentration of $Me_2NH{\cdot}BH_3$ (mM) for three different concentrations of catalyst **1** (28 mM, 22 mM, 15 mM). All reactions carried out at 60 °C in C_6D_6 with an initial substrate concentration of 0.29 M. All time values are given relative to an approximate start time, which is the time at which temperature ramping to 60 °C was begun. The linear correlation between υ and [$Me_2NH{\cdot}BH_3$] at high substrate concentrations is indicative of a first order dependence on substrate. However, the curvature at low substrate concentrations, and the fact that fitting the linear portion of the graph gives a non-zero y-intercept, indicates that the reaction is pseudo-first order with a more complex rate dependence at low substrate concentrations.

S4.3.2 – Procedure for determining activation parameters

Two stock solutions were prepared in the glovebox; a 600 mM solution of $Me_2NH{\cdot}BH_3$ in toluene, and a 9 mM solution of **1** in toluene. The two solutions were stored at −35 °C in the glovebox until required.

For each experiment, the stock solutions were warmed to room temperature and 250 µL of each solution was added to a Young's NMR tube, along with a sealed glass capillary of d_6-DMSO (to provide a lock). This afforded a reaction solution with an initial concentration of 0.30 M $Me_2NH{\cdot}BH_3$ and 4.5 mM **1** (1.5 mol% catalyst loading).

The sample was transferred to a Bruker AV(III) 600 spectrometer, preheated to the required reaction temperature (50 °C, 60 °C, 70 °C, or 80 °C), and the reaction temperature monitored by thermocouple.

For the reactions at 50 °C, 60 °C and 70 °C, the spectrometer was locked and shimmed to the d_6-DMSO, while for the reaction at 80 °C spectra were recorded locked but without shimming. ^{11}B NMR spectra were recorded at regular intervals until at least 80% of the starting material had been consumed. For all experiments, a T1 optimised ^{11}B NMR experiment was used (4 scans, 1 second pulse delay, 1–600 second delay between experiments). Conversion was quantified by integration of the ^{11}B NMR spectra, and absolute concentrations calculated from the known initial concentration of $Me_2NH{\cdot}BH_3$ (300 mM).

Rate constants were obtained by a linear fit of ln[$Me_2NH{\cdot}BH_3$] vs time for the first 3 half-lives of the reaction (until [$Me_2NH{\cdot}BH_3$] ≤ 37.5 mM, Figure S30). data from < 3 minutes after sample was transferred to the NMR machine were omitted for the runs at 80 °C as it was non-linear, likely due to temperature equilibration. Errors for the activation parameters obtained from the Eyring plot were estimated at three times the standard error calculated from the linear fit of the data.

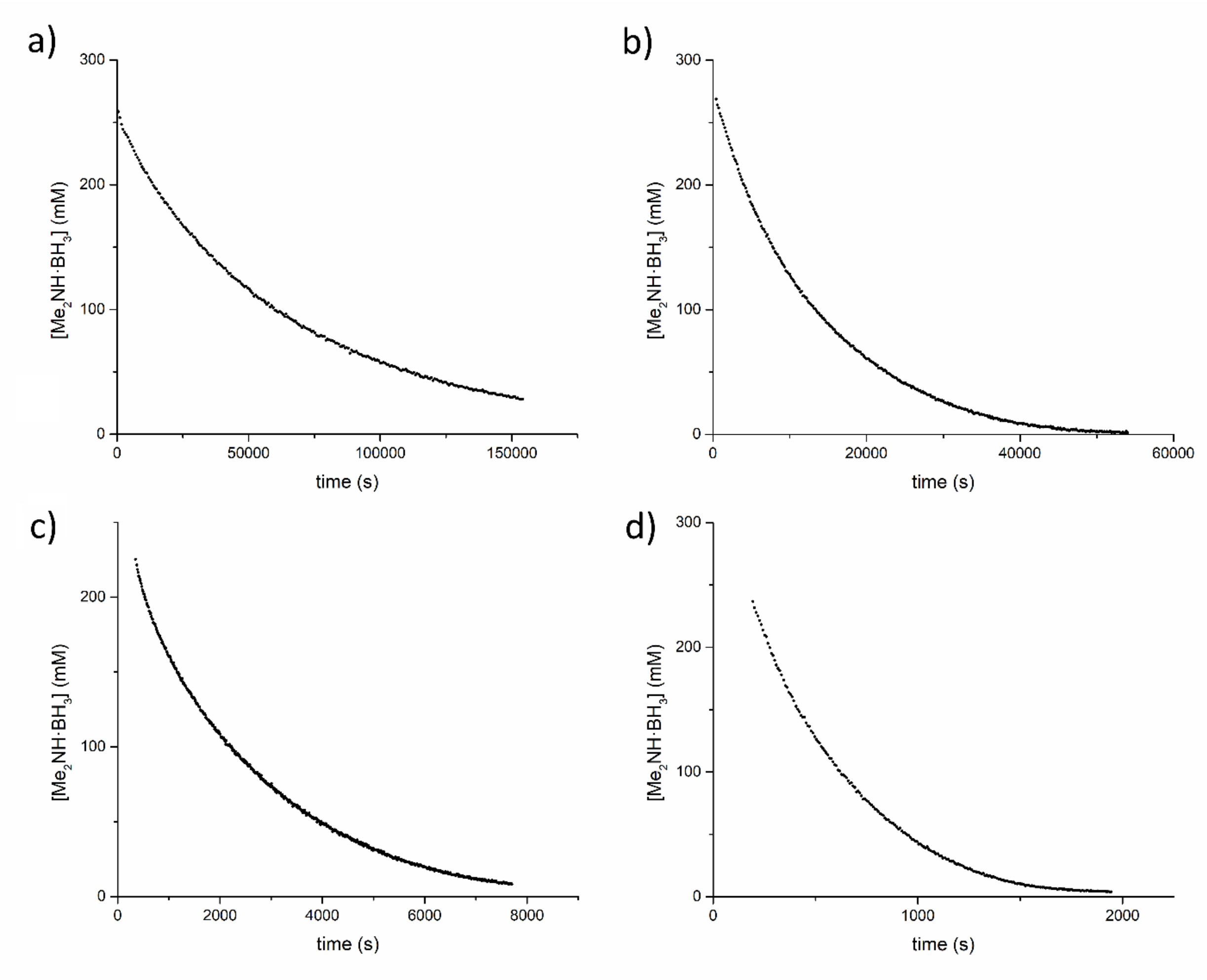

Figure S29: Plots of concentration of $Me_2NH{\cdot}BH_3$ (mM) vs time (s) for reactions at 50 °C (a), 60 °C (b), 70 °C (c), and 80 °C (d). All times are given relative to an approximate start point, which is when the sample was inserted into the NMR spectrometer. Concentration of **1** is 4.5 mM in all reactions.

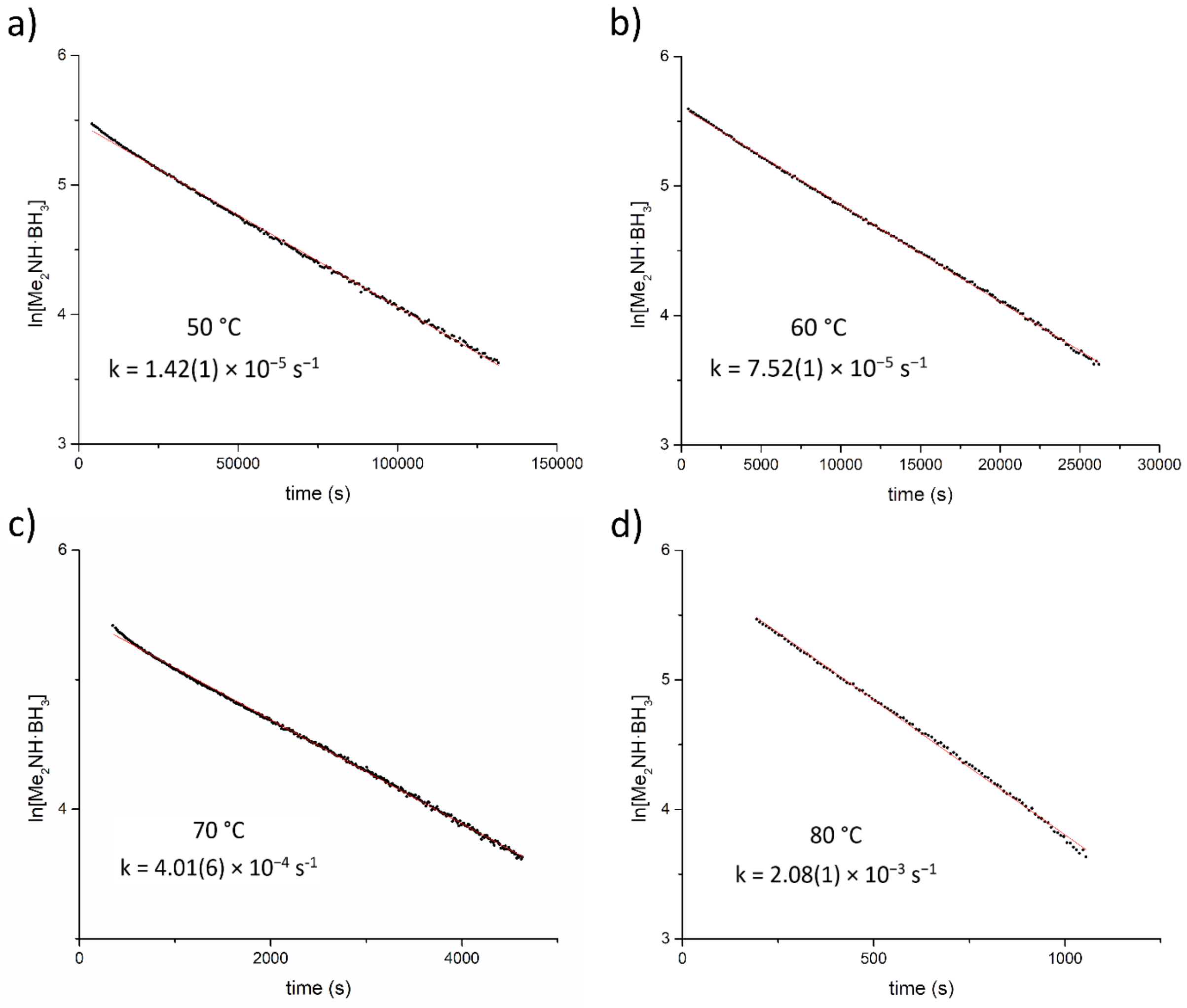

Figure S30: First order rate plots for the reactions at 50 °C (a), 60 °C (b), 70 °C (c), and 80 °C (d). Errors in rate constants are estimated from the standard error in the linear fitting of the data. All times are given relative to an approximate start point, which is when the sample was inserted into the NMR spectrometer. Concentration of **1** is 4.5 mM in all reactions.

S4.3.3 – Kinetic Isotope Effect with $Me_2ND{\cdot}BH_3$

In a Young's NMR tube 0.6 mL of a stock solution containing either $Me_2NH{\cdot}BH_3$ or $Me_2ND{\cdot}BH_3$ (0.29 M, see section S4.1.3) and catalyst **1** (22 mM) in C_6D_6 was prepared and frozen at −78 °C to prevent reaction initiation. The sample was transferred to a Bruker AV(III) 600 spectrometer preheated to 60 °C. The temperature was monitored using a thermocouple located within the probe. Once the temperature stabilised sufficiently to allow locking to the C_6D_6, alternating ^{11}B and ^{1}H NMR spectra were recorded at regular intervals until at least 95% of the starting material had been consumed. For all ^{11}B experiments, a T1 optimised ^{11}B NMR experiment was used (4 scans, 1 second pulse delay). Conversion was quantified by integration of the ^{11}B NMR spectra, and absolute concentrations calculated from the known initial concentration of $Me_2NH{\cdot}BH_3$ (290 mM). Pseudo-first order rate constants were obtained from plots of ln[S] ([S] = substrate concentration) *vs* time for the first 1700 s of the reaction.

$k_H = 6.8(1) \times 10^{-4}\ s^{-1}$

$k_D = 7.2(1) \times 10^{-4}\ s^{-1}$

These rate constants are within error of each other (*i.e.* 3 × standard error as determined by a linear fit of the data). Therefore $k_H/k_D \approx 1$

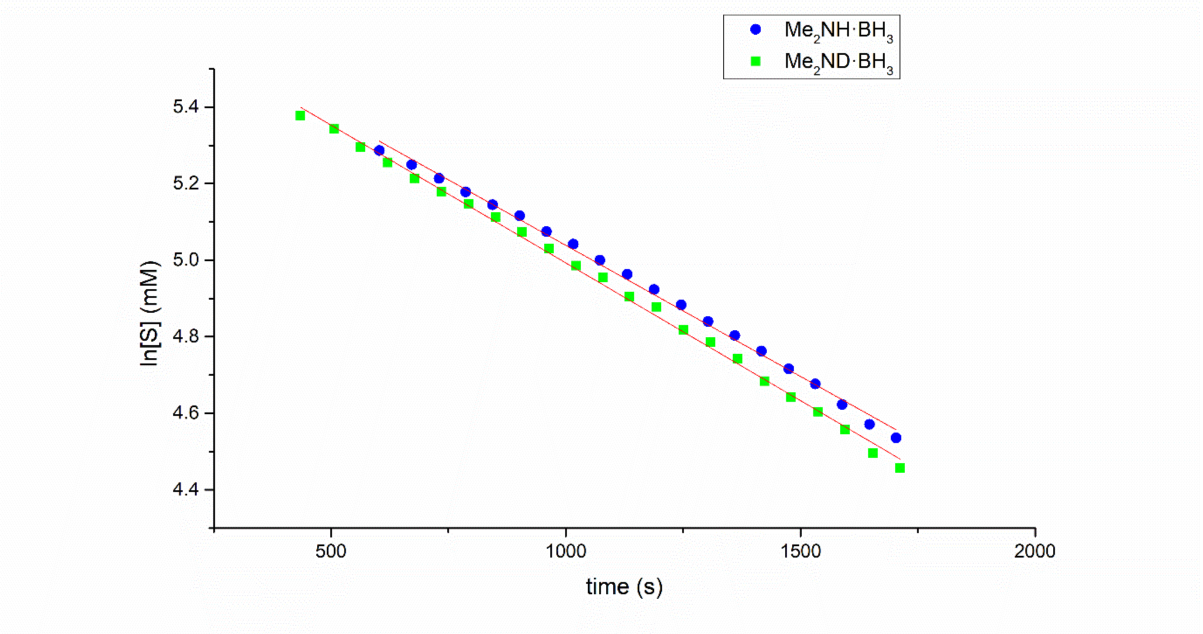

Figure S31: First order rate plot for reactions with $Me_2NH{\cdot}BH_3$ and $Me_2ND{\cdot}BD_3$

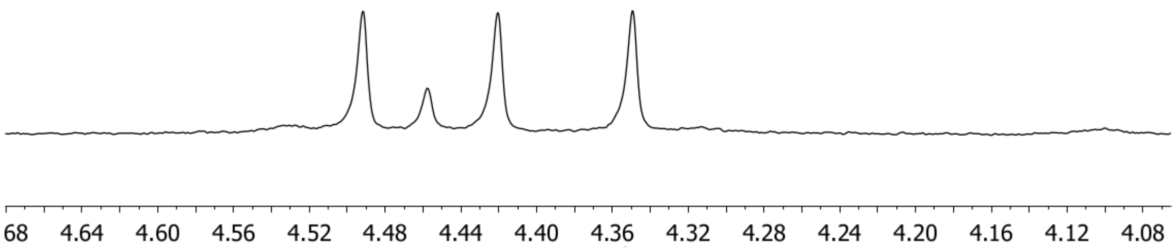

Figure S32: Signal from HD in ^{1}H NMR (600 MHz, 60 °C, C_6D_6) recorded during reaction with $Me_2ND{\cdot}BH_3$ as substrate

S4.3.3 – Kinetic Isotope Effect with $Me_2NH{\cdot}BD_3$

In a Young's NMR tube 0.6 mL of a stock solution containing a mixture of $Me_2NH{\cdot}BH_3$ and $Me_2NH{\cdot}BH_3$ (approximately 125 mM, see section S4.1.4), catalyst **1** (8.8 mM) and trimethoxybenzene (36 mM, for use as an internal standard) in C_6D_6 was prepared and frozen at −78 °C to prevent reaction initiation. The sample was transferred to a Bruker AV(III) 600 spectrometer and an initial ^1H NMR spectra recorded at 15 °C (Figure S33 and Figure S34). The sample was heated to 60 °C for 1 h, then cooled to 15 °C, whereupon a second ^1H NMR spectrum was recorded (Figure S33 and Figure S34). The methyl groups of $Me_2NH{\cdot}BH_3$ and $Me_2NH{\cdot}BD_3$ were integrated relative to the methyl groups of the trimethoxybenzene internal standard. Gaussian fitting was employed to deconvolute the peaks arising from $Me_2NH{\cdot}BH_3$ and $Me_2NH{\cdot}BD_3$. The kinetic isotope effect was calculated as k_H/k_D = 1.6 ± 0.1 from the equation:[14]

$$\frac{k_H}{k_D} = \frac{\ln\left(\frac{[\mathrm{Me_2NH \cdot BH_3}]_t}{[\mathrm{Me_2NH \cdot BH_3}]_0}\right)}{\ln\left(\frac{[\mathrm{Me_2NH \cdot BD_3}]_t}{[\mathrm{Me_2NH \cdot BD_3}]_0}\right)}$$

Where $[Me_2NH{\cdot}BH_3]_0$ and $[Me_2NH{\cdot}BD_3]_0$ are are the initial substrate concentrations, and $[Me_2NH{\cdot}BH_3]_t$ and $[Me_2NH{\cdot}BD_3]_t$ are the concentrations after heating for 1 h. ^1H NMR spectra were recorded with 8 scans, 1 dummy scan, pulse delay = 40 s, Acquisition time = 10.9 s. Spectra were processed with Gaussian window multiplication and a Fourier transform to improve signal resolution. T1 measurements: $Me_2NH{\cdot}BH_3/Me_2NH{\cdot}BD_3$ = 9.391 s, Trimethoxybenzene = 4.104 s.

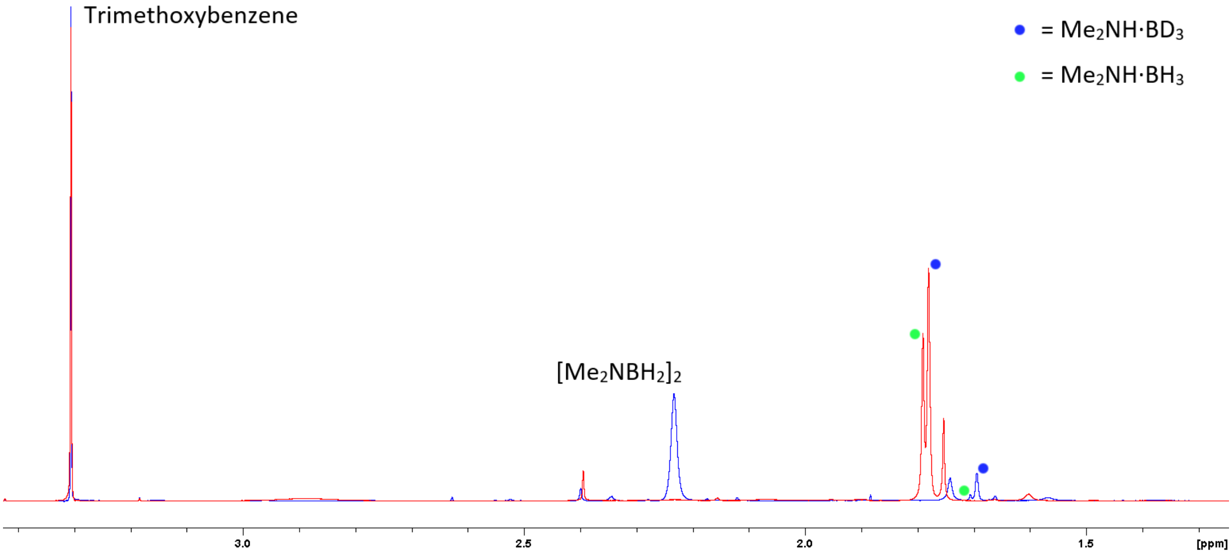

Figure S33: Superimposed ^1H NMR (600 MHz, 15 °C, C_6D_6) spectra showing sample before (red) and after (blue) heating Range 4.00–1.25 ppm.

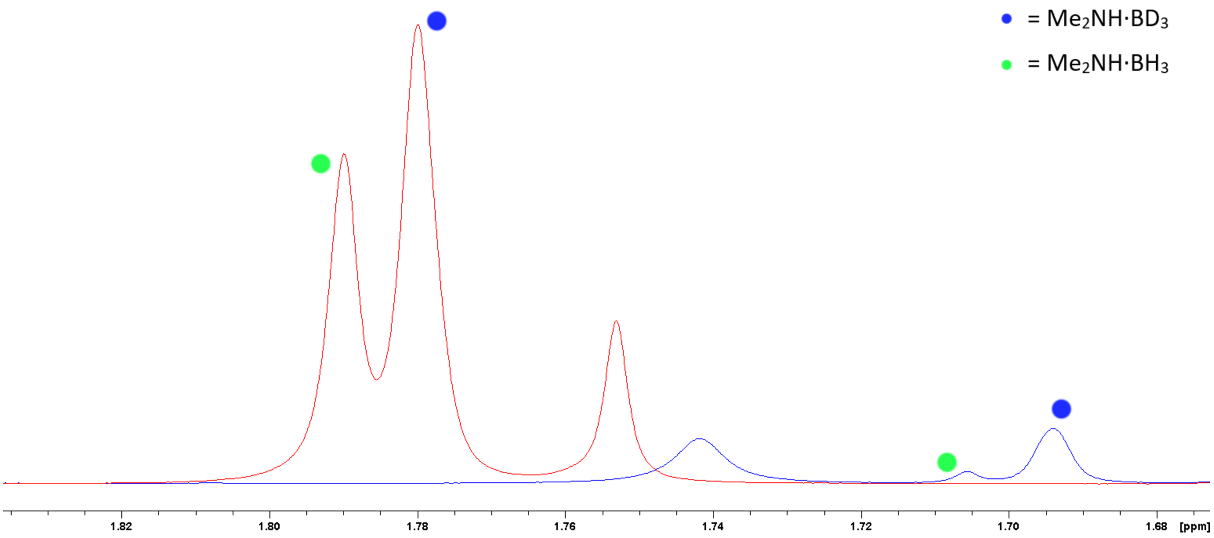

Figure S34: Superimposed ^1H NMR (600 MHz, 15 °C, C_6D_6) spectra showing sample before (red) and after (blue) heating. Range 1.84–1.67 ppm.

S4.4 – Stoichiometric Experiments

S4.4.1 – Reaction with $Me_2NH{\cdot}BH_3$

In a Young's NMR tube, $Me_2NH{\cdot}BH_3$ (2.0 mg, 0.034 mmol) and **1** (20.0 mg, 0.034 mmol) were dissolved in 0.8 mL of C_6D_6. The reaction was monitored by ^1H and ^{11}B NMR spectroscopy. The signals at δ_B 3.4 (t) and −14.6 (q) are proposed to correspond to species **I²** of the proposed catalytic cycle.

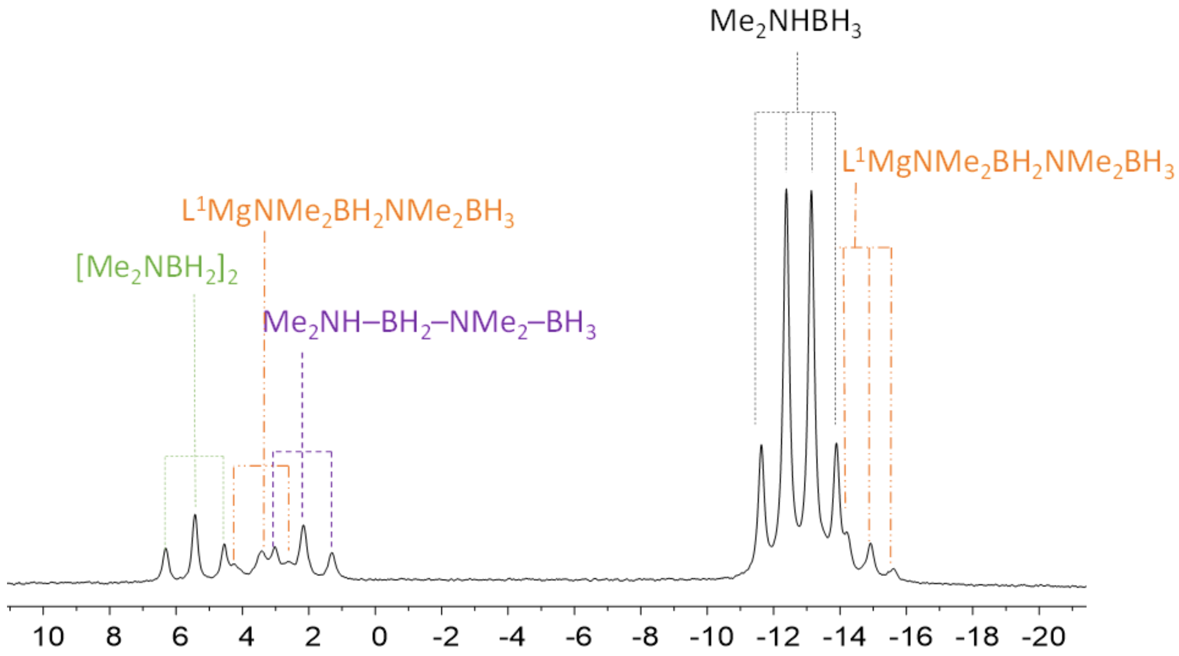

Figure S35: ^{11}B NMR spectrum for the stoichiometric reaction of 1 with 1 eq. of $Me_2HN{\cdot}BH_3$ in C_6D_6 after 44 h at room temperature. The signals at δ_B 3.4 (t) and −14.6 (q) are proposed to correspond to species **I²** of the proposed catalytic cycle.

S4.4.2 – Reaction with $Me_3N \cdot BH_3$

In a Young's NMR tube, $Me_3N \cdot BH_3$ (3.8 mg, 0.052 mmol) and **1** (15.0 mg, 0.026 mmol) were dissolved in 0.6 mL of C_6D_6. The reaction was heated to 80 °C and monitored by ^{1}H and ^{11}B NMR spectroscopies, whereupon no reaction was observed even after several days of heating.

S6 – References

[1] F. Ortu, G. J. Moxey, A. J. Blake, W. Lewis, D. L. Kays, *Inorg. Chem.* **2013**, *52*, 12429–12439.

[2] J. J. Klappa, A. E. Rich, K. McNeill, *Org. Lett.* **2002**, *4*, 435–437.

[3] J. Cosier, A. M. Glazer, *J. Appl. Cryst.* **1986**, *19*, 105–107.

[4] Agilent Technologies, **2013**.

[5] G. M. Sheldrick, *Acta Crystallogr. A* **2008**, *64*, 112–122.

[6] O. V Dolomanov, L. J. Bourhis, R. J. Gildea, J. A. K. Howard, H. Puschmann, *J. Appl. Cryst.* **2009**, *42*, 339–341.

[7] O. V Dolomanov, A. J. Blake, N. R. Champness, M. Schröder, *J. Appl. Cryst.* **2003**, *36*, 1283–1284.

[8] CrysAlisPRO Oxford Diffraction Agilent Technologies UK Ltd.

[9] G. M. Sheldrick, *Acta Crystallogr. C* **2015**, *71*, 3–8.

[10] G. M. Sheldrick, *Acta Crystallogr. A* **2015**, *71*, 3–8.

[11] “CheckCIF,” can be found under http://checkcif.iucr.org

[12] W. H. Myers, G. E. Ryschkewitsch, *Inorg. Chem.* **1978**, *17*, 1157–1159.

[13] N. T. Coles, M. F. Mahon, R. L. Webster, *Organometallics* **2017**, *36*, 2262–2268.

[14] L. Melander, W. H. Saunders, *Reaction Rates of Isotopic Molecules*, John Wiley & Sons, Ltd., **1960**.
